# Supplementary material for: A hidden gene in astroviruses encodes a viroporin
Source: Nat Commun. 2020 Aug 13;11:4070. doi: 10.1038/s41467-020-17906-x (PMC7426862; doi:10.1038/s41467-020-17906-x)
Supplement: Supplementary file 1 — Supplementary Information [file 41467_2020_17906_MOESM1_ESM.pdf]

A hidden gene in astroviruses encodes a viroporin

Lulla *et al.*

Supplementary information

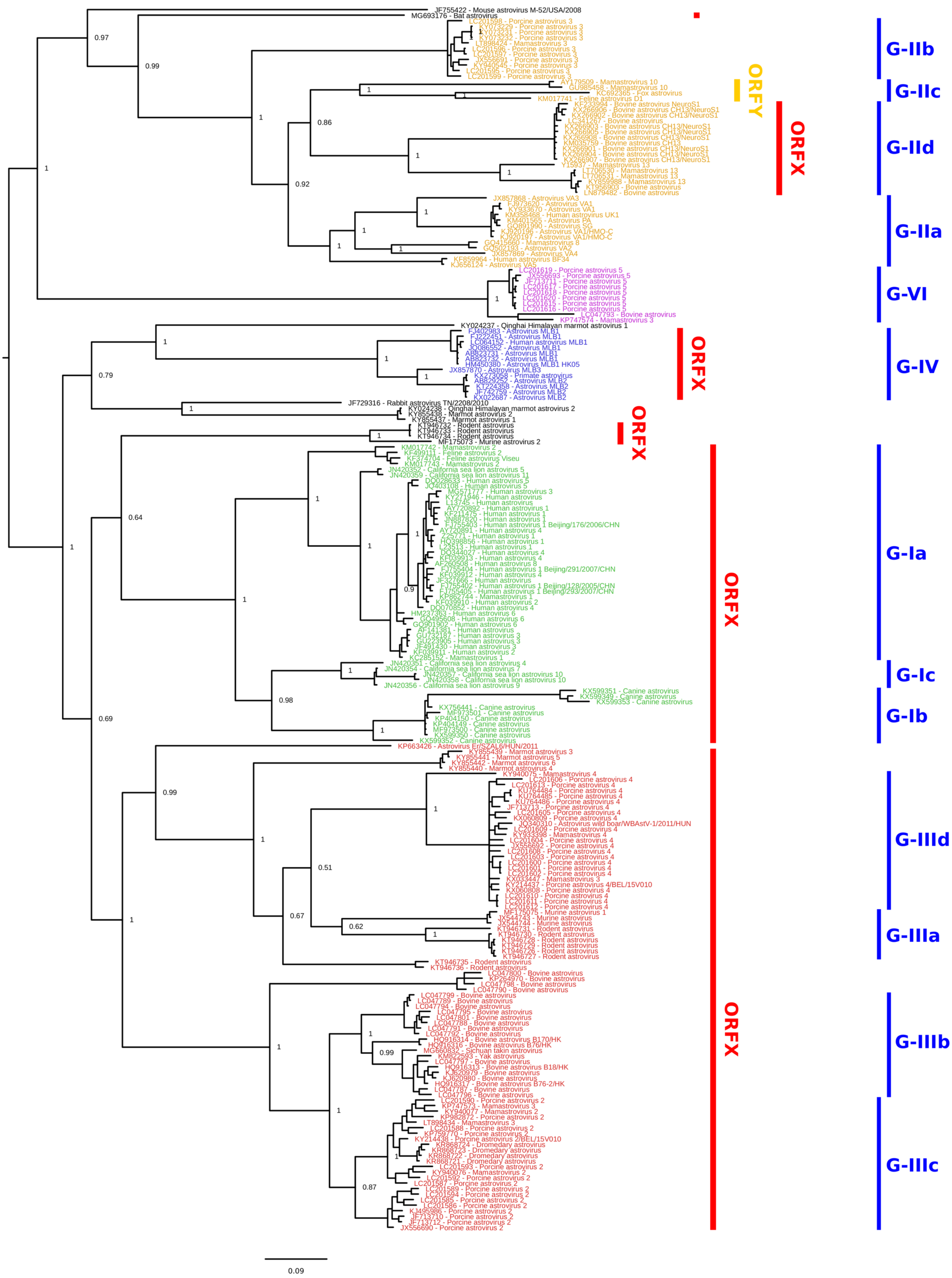

Supplementary Figure 1 | Phylogenetic tree of mammalian astrovirus RdRp sequences. All full-length mammalian astrovirus genome sequences were obtained from NCBI on 26 July 2018 and the ORF1b amino acid sequences were extracted and aligned with MUSCLE[1]. A maximum likelihood phylogenetic tree was estimated using the Bayesian Markov chain Monte Carlo method implemented in MrBayes version 3.2.3[2], sampling across the default set of fixed amino acid rate matrices, with one

million generations, discarding the first 25% as burn-in. The tree was visualized with FigTree (http://tree.bio.ed.ac.uk/software/figtree/). Genogroups (based on the scheme of Yokoyama et al[3]) are indicated with coloured text: green – genogroup I, yellow – genogroup II, red – genogroup III, blue – genogroup IV, magenta – genogroup VI, unclassified sequences – black. Subgroups of sequences, defined on the basis of RdRp phylogeny for the purposes of this study only, are indicated in blue at right (G-Ia, G-Ib, etc). Taxa that have a putative ORFX (ORFY) are indicated in red (yellow) at right. The tree is midpoint rooted and deeper nodes are labelled with posterior probability values.

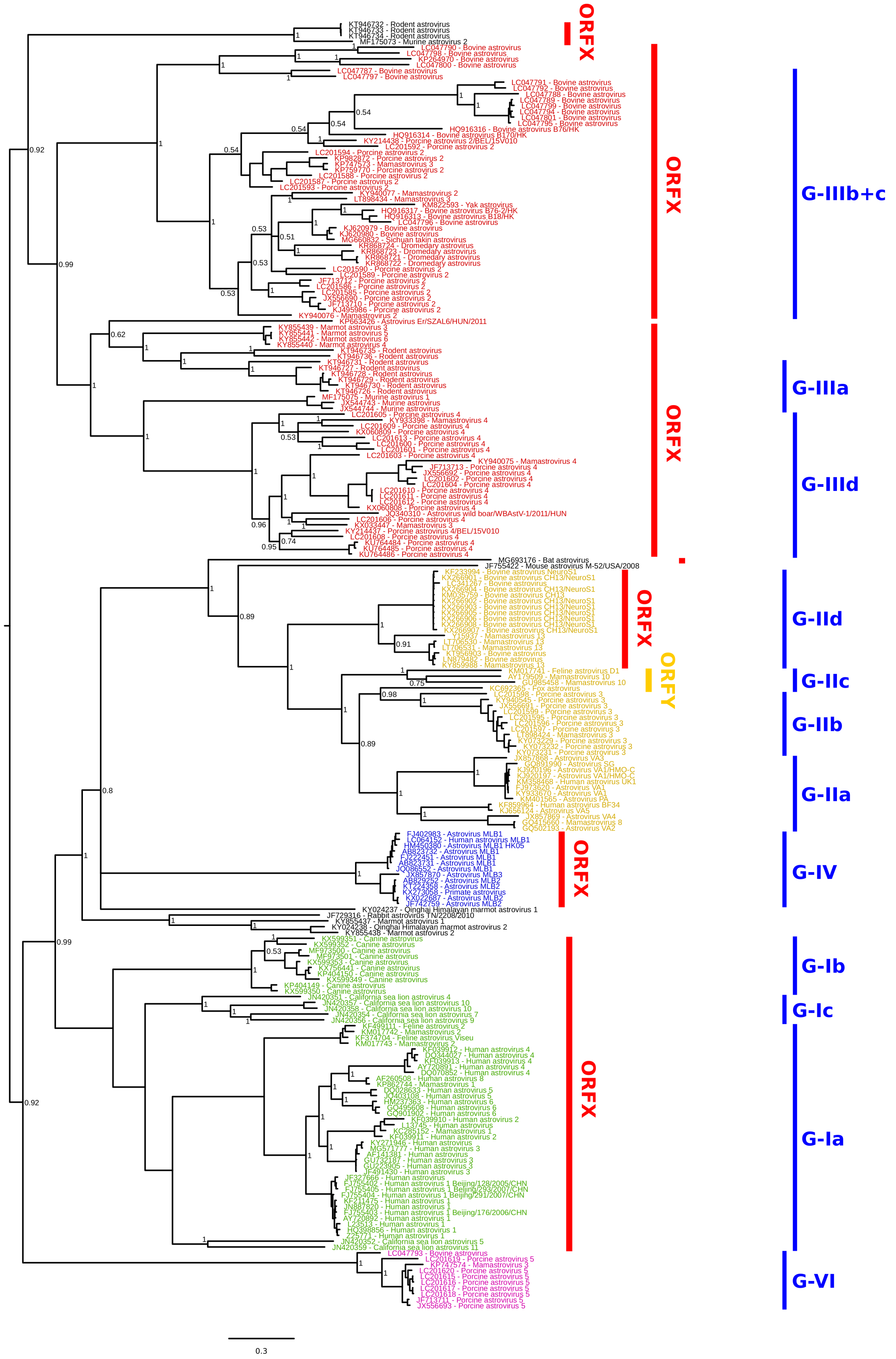

Supplementary Figure 2 │ Phylogenetic tree of mammalian astrovirus CP sequences. All full-length mammalian astrovirus genome sequences were obtained from NCBI on 26 July 2018 and the

ORF2 amino acid sequences were extracted and aligned with MUSCLE[1]. A maximum likelihood phylogenetic tree was estimated using the Bayesian Markov chain Monte Carlo method implemented in MrBayes version 3.2.3[2], sampling across the default set of fixed amino acid rate matrices, with one million generations, discarding the first 25% as burn-in. The tree was visualized with FigTree (http://tree.bio.ed.ac.uk/software/figtree/). Genogroups (based on the scheme of Yokoyama et al[3]) are indicated with coloured text: green – genogroup I, yellow – genogroup II, red – genogroup III, blue – genogroup IV, magenta – genogroup VI, unclassified sequences – black. Subgroups of sequences, defined on the basis of RdRp phylogeny (Supplementary Fig. 1) for the purposes of this study only, are indicated in blue at right (G-Ia, G-Ib, etc). Taxa that have a putative ORFX (ORFY) are indicated in red (yellow) at right. The tree is midpoint rooted and deeper nodes are labelled with posterior probability values.

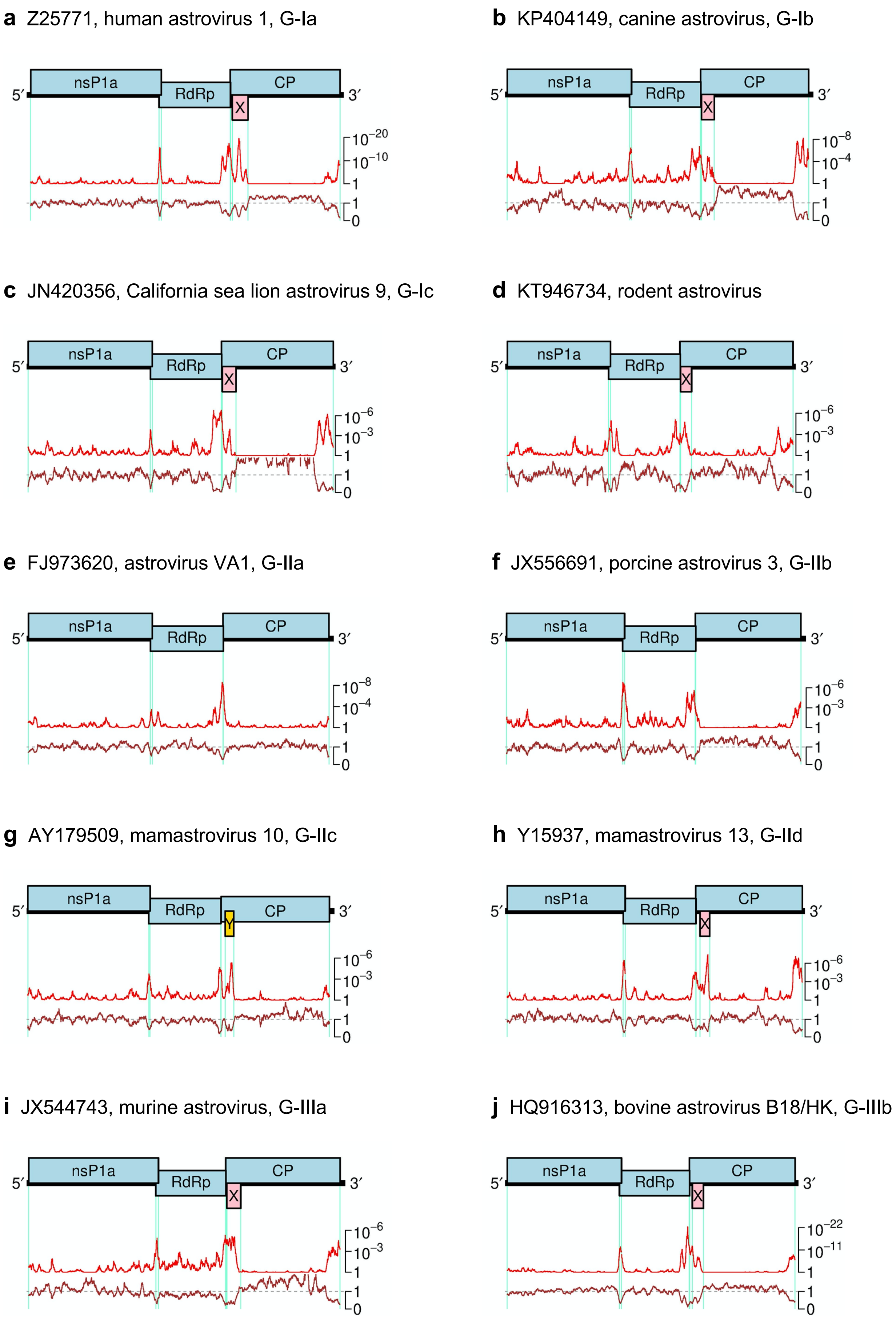

a Z25771, human astrovirus 1, G-Ia
b KP404149, canine astrovirus, G-Ib
c JN420356, California sea lion astrovirus 9, G-Ic
d KT946734, rodent astrovirus
e FJ973620, astrovirus VA1, G-IIa
f JX556691, porcine astrovirus 3, G-IIb
g AY179509, mamastrovirus 10, G-IIc
h Y15937, mamastrovirus 13, G-IId
i JX544743, murine astrovirus, G-IIIa
j HQ916313, bovine astrovirus B18/HK, G-IIIb
nsP1a
RdRp
CP
X
Y
5′
3′
10^{-20}
10^{-10}
10^{-8}
10^{-4}
10^{-6}
10^{-3}
10^{-22}
10^{-11}
1
0

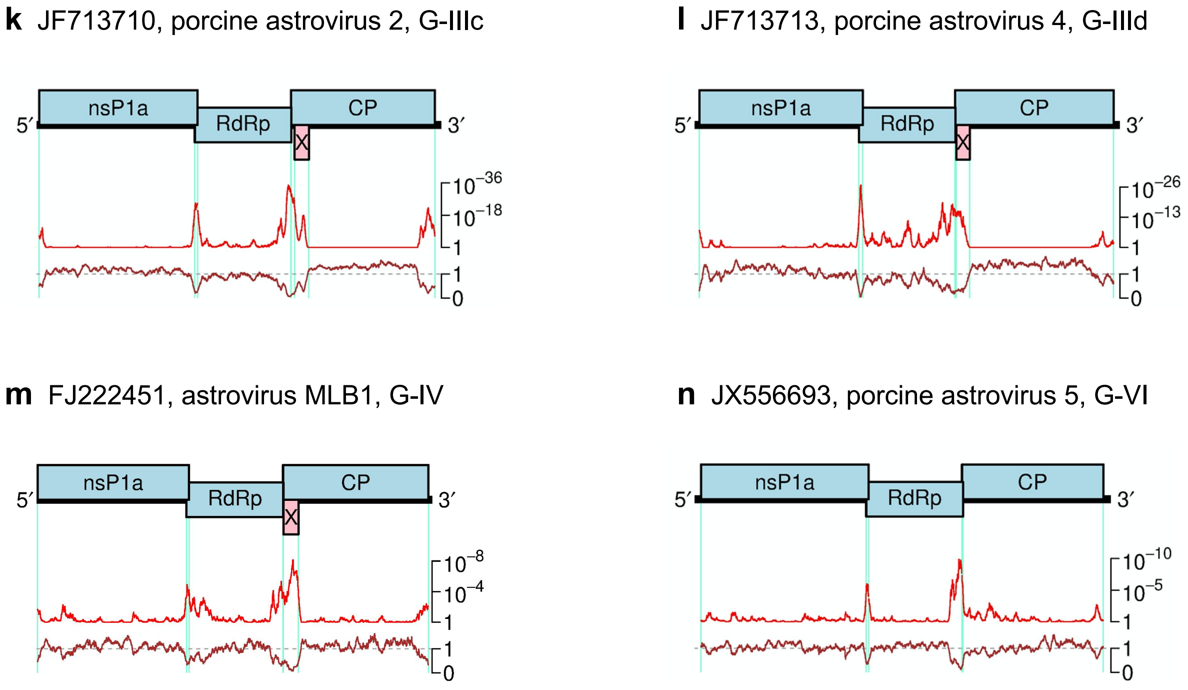

Supplementary Figure 3 | Synonymous site conservation analysis of astroviruses. In each subfigure, a genome map is shown at top, indicating the nsP1a, RdRp and CP ORFs (blue) and the putative additional ORF where present (pink – ORFX; yellow – ORFY). Below, is shown the analysis of conservation at synonymous sites in the nsP1a, RdRp and CP ORFs. The red line shows the probability that the observed conservation could occur under a null model of neutral evolution at synonymous sites, whereas the brown line depicts the ratio of the observed number of substitutions to the number expected under the null model. Peaks in synonymous site conservation may indicate functionally important overlapping elements such as the −1 PRF signal between the nsP1a and RdRp ORFs, sgRNA promoter sequences, and overlapping coding sequences (i.e. the putative X and Y ORFs). Each synonymous site conservation analysis is based on an alignment of virus sequences in the indicated clade (Ia, Ib, Ic, etc) using the genome coordinate system of the indicated reference sequence / isolate name. The subgroup designations are defined in Fig. 1b and the sequences used in each alignment are as shown in Supplementary Fig. 9.

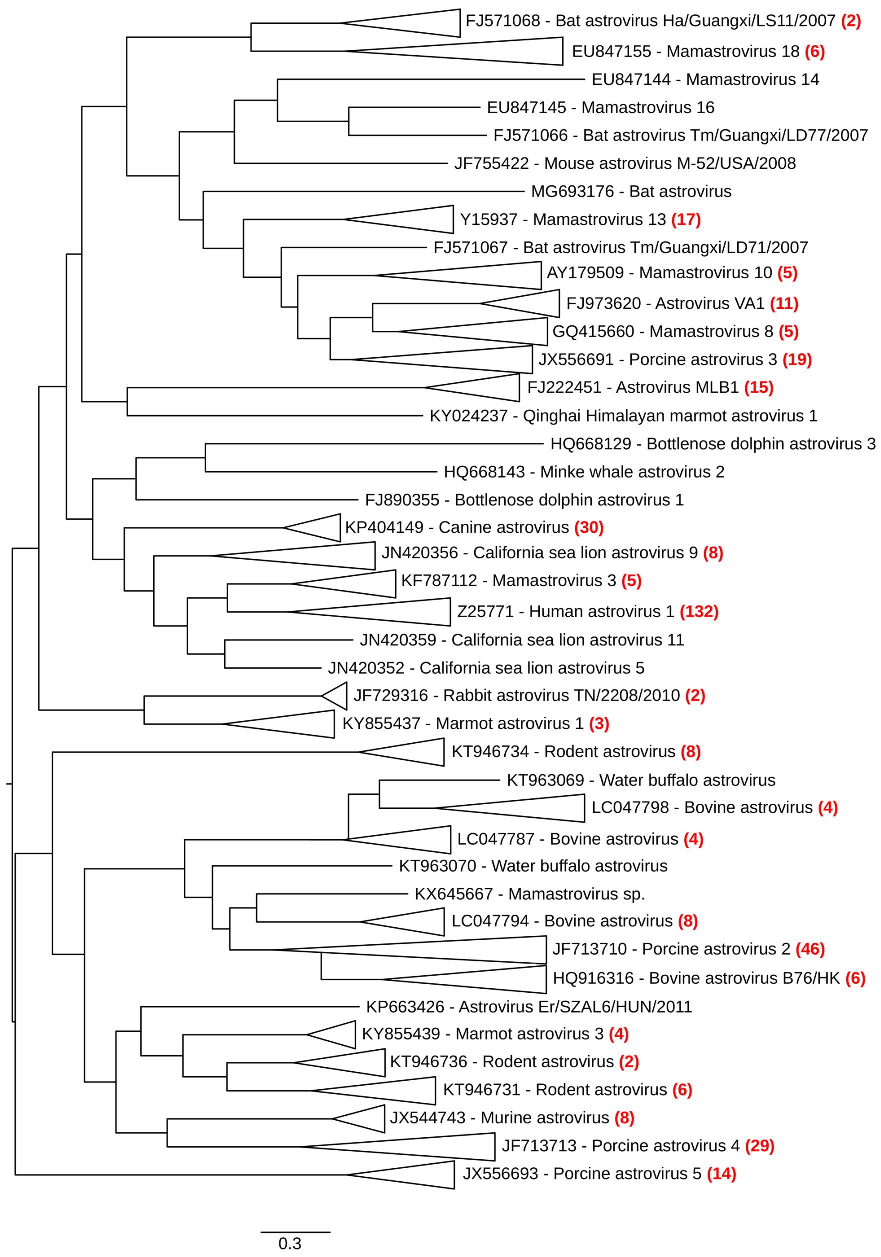

Supplementary Figure 4 | Phylogenetic tree of mammalian astrovirus CP sequences. Mammalian astrovirus sequences with complete or nearly complete coverage of ORF2 were obtained from NCBI on 26 July 2018, and the ORF2 amino acid sequences were extracted and aligned with MUSCLE[1]. A maximum likelihood phylogenetic tree was estimated using the Bayesian Markov chain Monte Carlo method implemented in MrBayes version 3.2.3[2], sampling across the default set of fixed amino acid rate matrices, with five million generations, discarding the first 25% as burn-in. The tree was midpoint rooted and visualized with FigTree (http://tree.bio.ed.ac.uk/software/figtree/). Related groups of sequences (indicated by isosceles triangles) have been replaced in the figure by a single representative accession number and virus name; the total number of sequences in each group is shown in red. The 26 groups and 16 singletons correspond to those used in Supplementary Fig. 5 and Supplementary Fig. 7, respectively. The complete list of 415 sequences is shown in Supplementary Table 2.

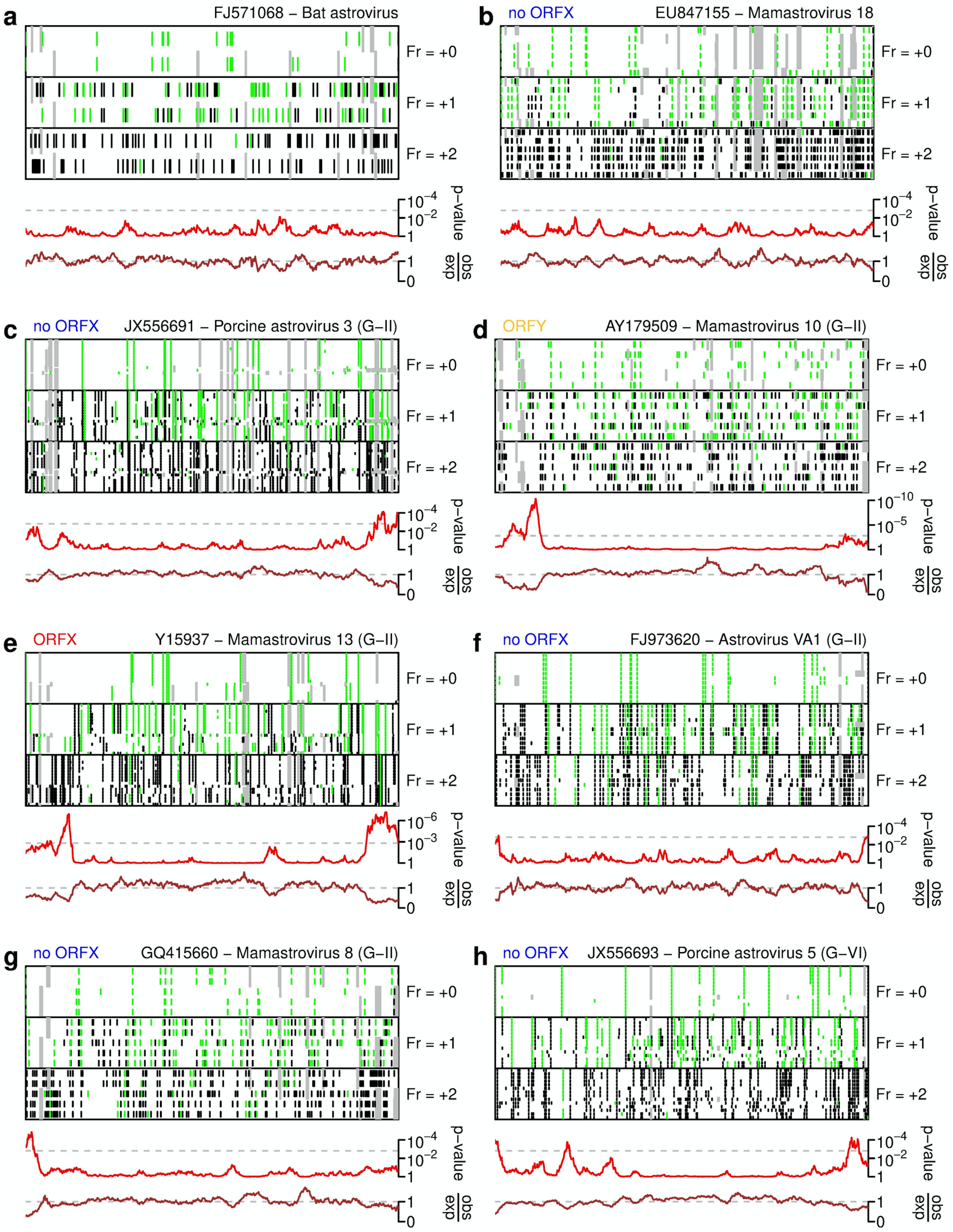
a
FJ571068 – Bat astrovirus
b
no ORFX
EU847155 – Mamastrovirus 18
c
no ORFX
JX556691 – Porcine astrovirus 3 (G-II)
d
ORFY
AY179509 – Mamastrovirus 10 (G-II)
e
ORFX
Y15937 – Mamastrovirus 13 (G-II)
f
no ORFX
FJ973620 – Astrovirus VA1 (G-II)
g
no ORFX
GQ415660 – Mamastrovirus 8 (G-II)
h
no ORFX
JX556693 – Porcine astrovirus 5 (G-VI)
Fr = +0
Fr = +1
Fr = +2
p-value
obs/exp

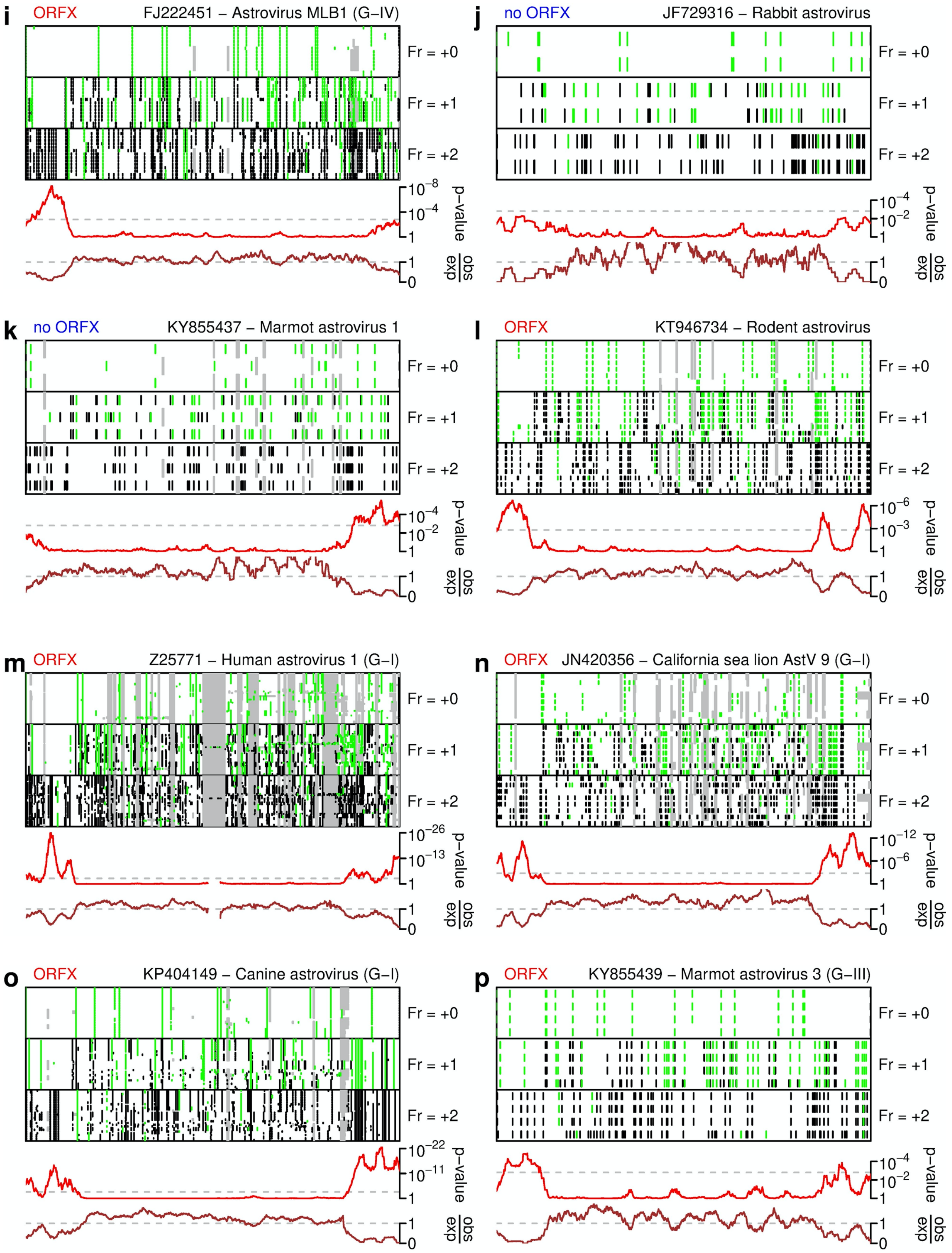
i
ORFX
FJ222451 – Astrovirus MLB1 (G–IV)
Fr = +0
Fr = +1
Fr = +2
p–value
obs/exp
j
no ORFX
JF729316 – Rabbit astrovirus
k
no ORFX
KY855437 – Marmot astrovirus 1
l
ORFX
KT946734 – Rodent astrovirus
m
ORFX
Z25771 – Human astrovirus 1 (G–I)
n
ORFX
JN420356 – California sea lion AstV 9 (G–I)
o
ORFX
KP404149 – Canine astrovirus (G–I)
p
ORFX
KY855439 – Marmot astrovirus 3 (G–III)

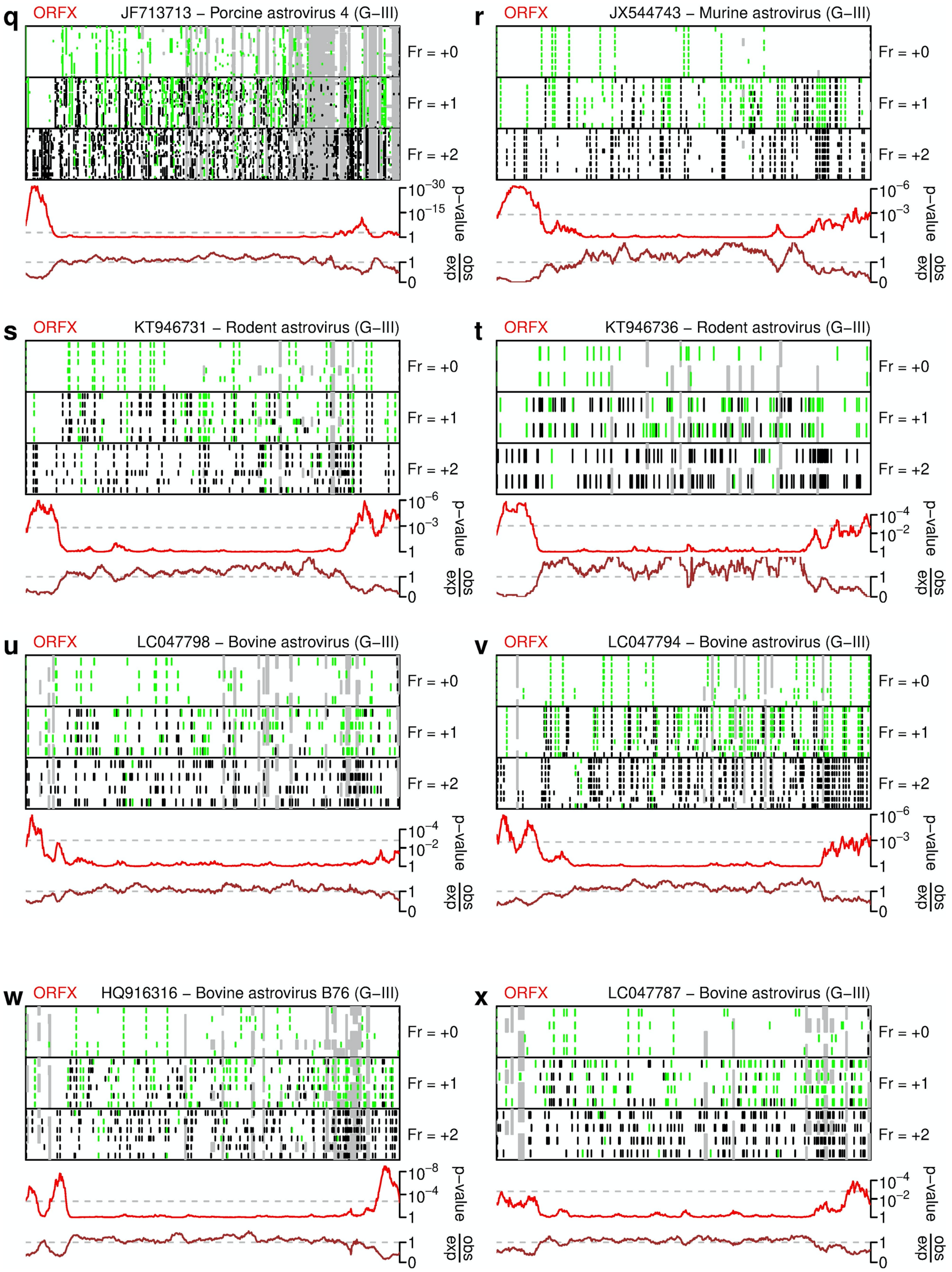

q ORFX JF713713 – Porcine astrovirus 4 (G–III)
r ORFX JX544743 – Murine astrovirus (G–III)
s ORFX KT946731 – Rodent astrovirus (G–III)
t ORFX KT946736 – Rodent astrovirus (G–III)
u ORFX LC047798 – Bovine astrovirus (G–III)
v ORFX LC047794 – Bovine astrovirus (G–III)
w ORFX HQ916316 – Bovine astrovirus B76 (G–III)
x ORFX LC047787 – Bovine astrovirus (G–III)
Fr = +0
Fr = +1
Fr = +2
p–value
obs/exp

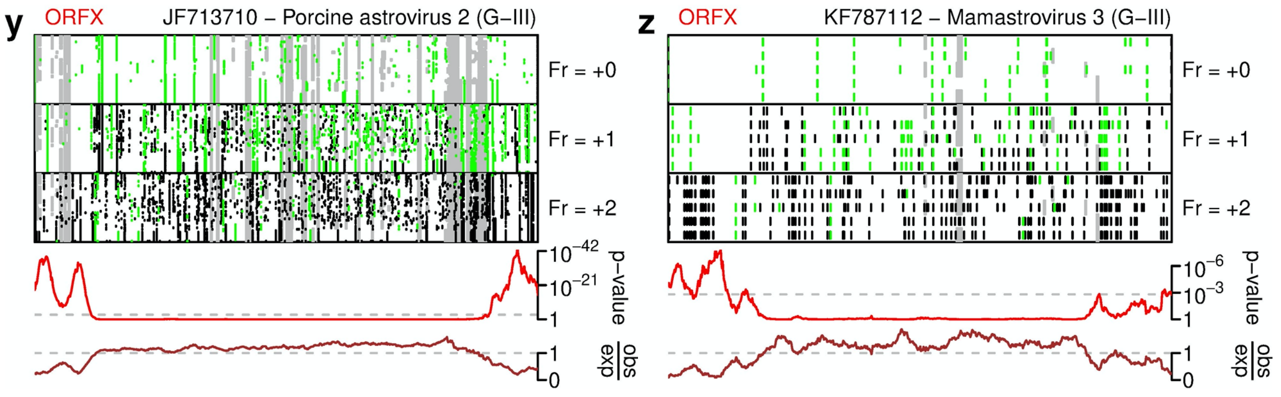

Supplementary Figure 5 | Comparative genomic analysis of CP ORF alignments. Sequences with coverage of the capsid coding region of mammalian astroviruses were obtained from NCBI, CP ORF sequences were extracted, and clustered into 26 multi-sequence and 16 singleton groups based on CP amino acid identity (see Methods; Supplementary Fig. 4). For each multi-sequence group, duplicate sequences were removed and remaining sequences were aligned with MUSCLE[1] and analysed with synplot2[4]. The header of each plot indicates one sequence (accession number and virus name) from the group, genogroup (G-I, G-II etc; where defined), and whether the group is predicted to contain ORFX, ORFY or neither. The upper three panels show the positions of alignment gaps (grey), stop codons (black) and AUG codons (green) in each of the three reading frames in each sequence in the alignment. Below, is shown the analysis of conservation at synonymous sites. The red line shows the probability that the observed conservation could occur under a null model of neutral evolution at synonymous sites, whereas the brown line depicts the ratio of the observed number of substitutions to the number expected under the null model. Peaks in synonymous site conservation may indicate functionally important overlapping elements such as overlapping coding sequences (i.e. the putative X and Y ORFs) besides regulatory elements (e.g. functional RNA structures). See Supplementary Fig. 7 for the singleton groups.

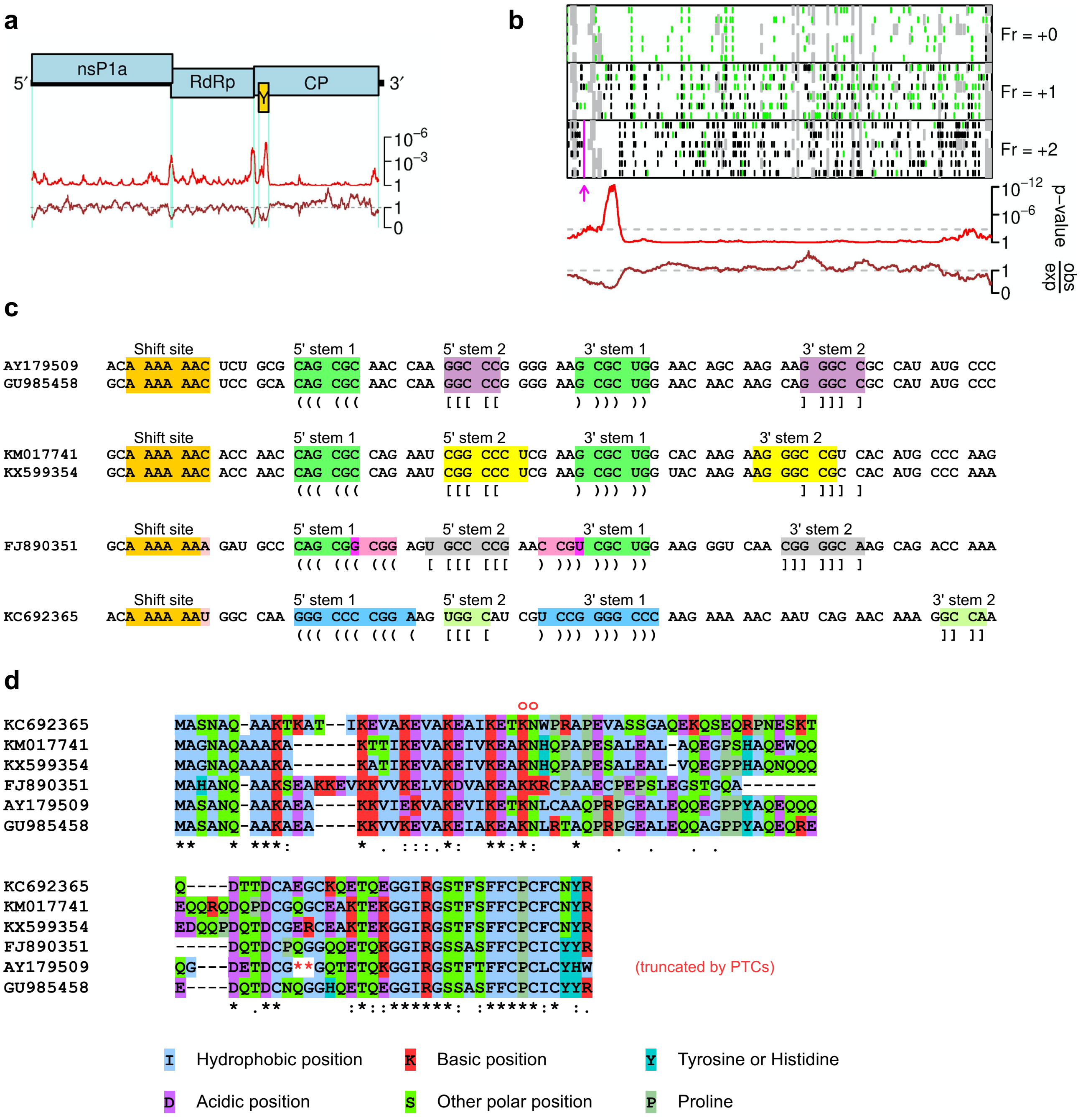

Supplementary Figure 6 | A putative −1 PRF overlapping ORF in subgroup IIc astroviruses. (a) Comparative genomic analysis of subgroup IIb full-length sequences AY179509, GU985458, KC692365 and KM017741. Top – genome map indicating the nsP1a, RdRp and CP ORFs (blue) and the putative additional Y ORF (yellow). Bottom – analysis of conservation at synonymous sites in the nsP1a, RdRp and CP ORFs. The red line shows the probability that the observed conservation could occur under a null model of neutral evolution at synonymous sites, whereas the brown line depicts the ratio of the observed number of substitutions to the number expected under the null model. Peaks in synonymous site conservation may indicate functionally important overlapping elements such as the −1 ribosomal frameshifting signal between the nsP1a and RdRp ORFs, sgRNA promoter sequences, and overlapping coding sequences (i.e. the putative Y ORF). **(b)** Synonymous site conservation plot for the CP ORF for AY179509, GU985458, KC692365, KM017741 and two additional partial-genome sequences with CP ORF coverage, FJ890351 and KX599354 (lower two panels). In the upper three panels, alignment gaps (grey), stop codons (black) and AUG codons (green) are indicated in each of the three reading frames. The location of the putative A_AAA_AAZ −1 ribosomal frameshifting site is indicated in pink. **(c)** Predicted −1 PRF shift sites and 3′-adjacent

RNA secondary structures in each of the six sequences. Predicted base-pairings are indicated with matching highlights and "()"s (stem 1) or "[]"s (stem 2). **(d)** Alignment of the predicted −1 PRF product. The location of the A_AAA_AAN shift site is indicated with two red circles. The upstream sequence is encoded by the 5′ end of the CP ORF whereas the downstream sequence is encoded by the overlapping −1 frame Y ORF. Amino acids are colour-coded according to their physicochemical properties. In AY179509, the Y ORF is truncated by two tandem premature termination codons (red asterisks).

EU847145 - Mamastrovirus 16

MRLEINLTKRISTRVSKSMLGRRLRRKRGRMVMVDLNLNLLLMLLLLLGLLRAVMSLL
???????????????????????

FJ890355 - Bottlenose dolphin astrovirus 1

MLALRLKPPAHREAKVVPVQGLEEEHPLSKSQLIPKQKDLPEDQVDALLELKIIVSNNKLEINSRNKVSQGPPQRLSRPRRLLLALLDQILAMMQRGRFPSI
??????????????????

HQ668129 - Bottlenose dolphin astrovirus 3

MAAKSQLKSSPEPELRLVKAITRELVEEEKEVIRTIKRMLANNQEIIITRLGVSEQRYHVQLTAWAYADPNQKIKSSSLQCLVPSVETHQEGSKWRRWFSLTRCSLRKSQGITT

LDHCK

HQ668143 - Minke whale astrovirus 2

MPHNLKRNASVSGVTQEELELLEILTAILESHDEVIVDVINRMEGRLELRLNAWDSEGLSQHSHRLSRPRWAPLVPMRRLFLRLKGFFTSTPHSLRRSPGVWRLDRFRP

JN420352 - California sea lion astrovirus 5

MAATGANLVPAPRVGAGNQMSRSQSIQNPEEQEVELDAVNVNLITVSVNLSNNSLTSLVLQDQSQPSDKKPLLRLALSTLIRLIRRSLRR
?????????????????

JN420359 - California sea lion astrovirus 11

MKNPTGAILAAAHNPEAATRQSRSPSIQRSQEVETDAVNVNLVSVSVQLSINNSGNRVSQDQKCQLNSGQQQHSEPLEPTPLGSQSLRRASFVTRFLSRTVQVLRLLAQFRC

KT963069 - Water buffalo astrovirus

MAMGGMPRAWWFRPLRVTNRHRTKVDGVDGARLRSMSTSQTRQDHQEDQQDNNNNLRDNVNFGCGATAALRAVKLRFSIRYARR
??????????????????

KT963070 - Water buffalo astrovirus

MDQHPQEQVDDVDGVEPNPENPKLLCCLPLRKFRFRVSGPAVRASITMWFGRESPPPLEQLEPTPTGK

KX645667 - Mamastrovirus sp.

MEDQHNPPPNPSRDETDGRDIMRRLRRLYALYKELKNVADQILQEDVVEGGLYCRKLQQRSEQLDQMLQKQSKTSSQSSSIPPP

MG693176 - Bat astrovirus

MPSLRTRRLRLRSRLNLISNILRNTNRNRPRARVRLEQSLRRLRRTLRRSVLRGRNRGRQSVSRPLSASLTGPPRRARCWLLVQIFILLLPRNLKVALLSALWLRRRPSLANGA

FLGLLSVLPPWLALLLLLALSLAFPSIQQAGPFQ

Supplementary Figure 7 | Potential XP sequences in unclustered astroviruses. Sequences with coverage of the capsid coding region of mammalian astroviruses were obtained from NCBI, CP ORF sequences were extracted, and clustered into 26 multi-sequence and 16 singleton groups based on CP amino acid identity (see Methods). Potential XP peptides encoded within 10 of the 16 unclustered sequences are shown. Amino acids are colour-coded according to their physicochemical properties. Transmembrane regions predicted by Phobius[5] are indicated with pink bars. Potential transmembrane regions (hydrophobic regions scored below threshold by Phobius) are indicated with pink question marks. See Supplementary Fig. 5 for the multi-sequence groups.

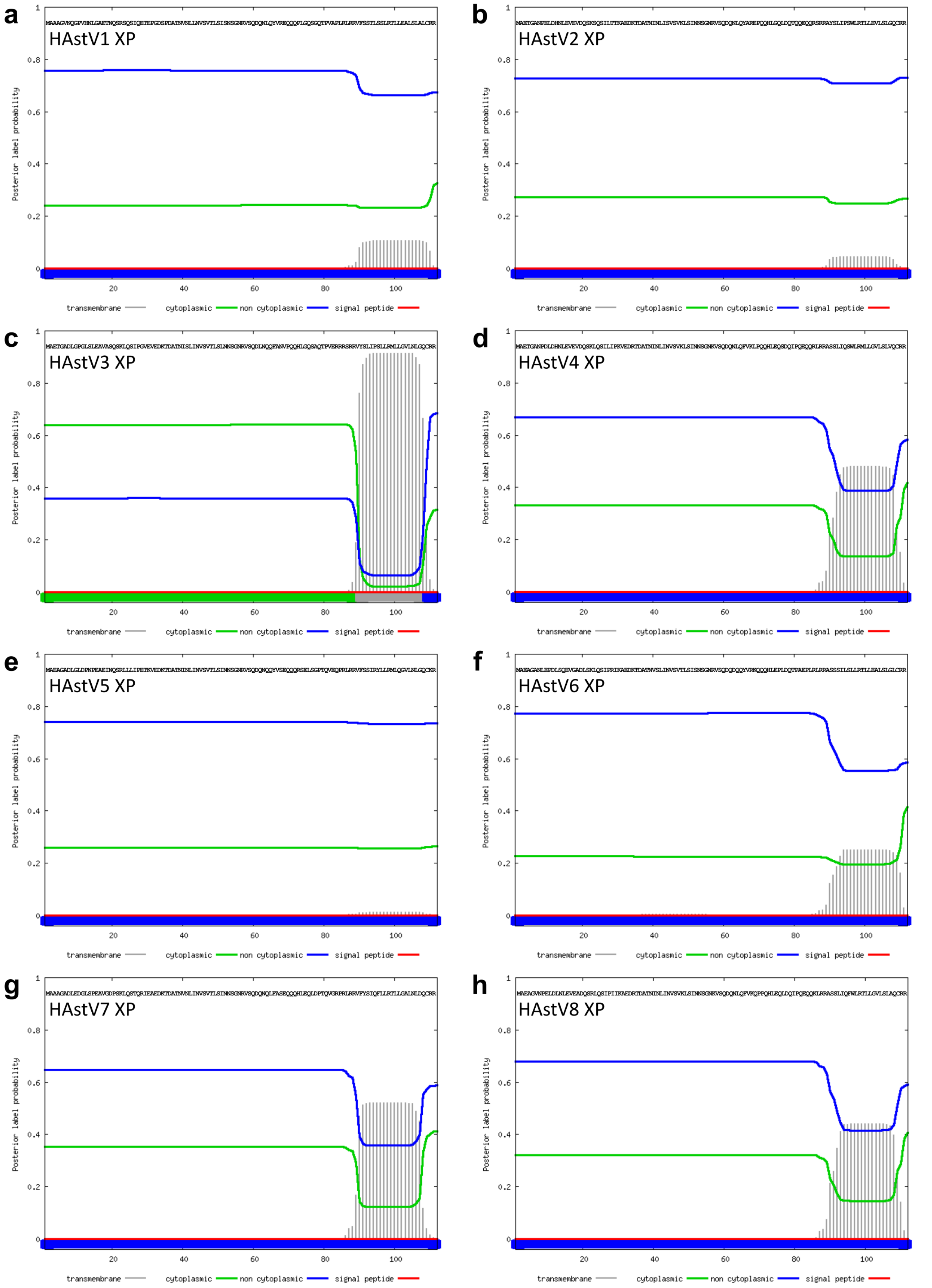

Supplementary Figure 8 | Transmembrane domain predictions for HAstV XPs. Transmembrane domains for representative sequences (see Fig. 1f) were predicted with Phobius[5]. An above-threshold TM was predicted only for HAstV3.

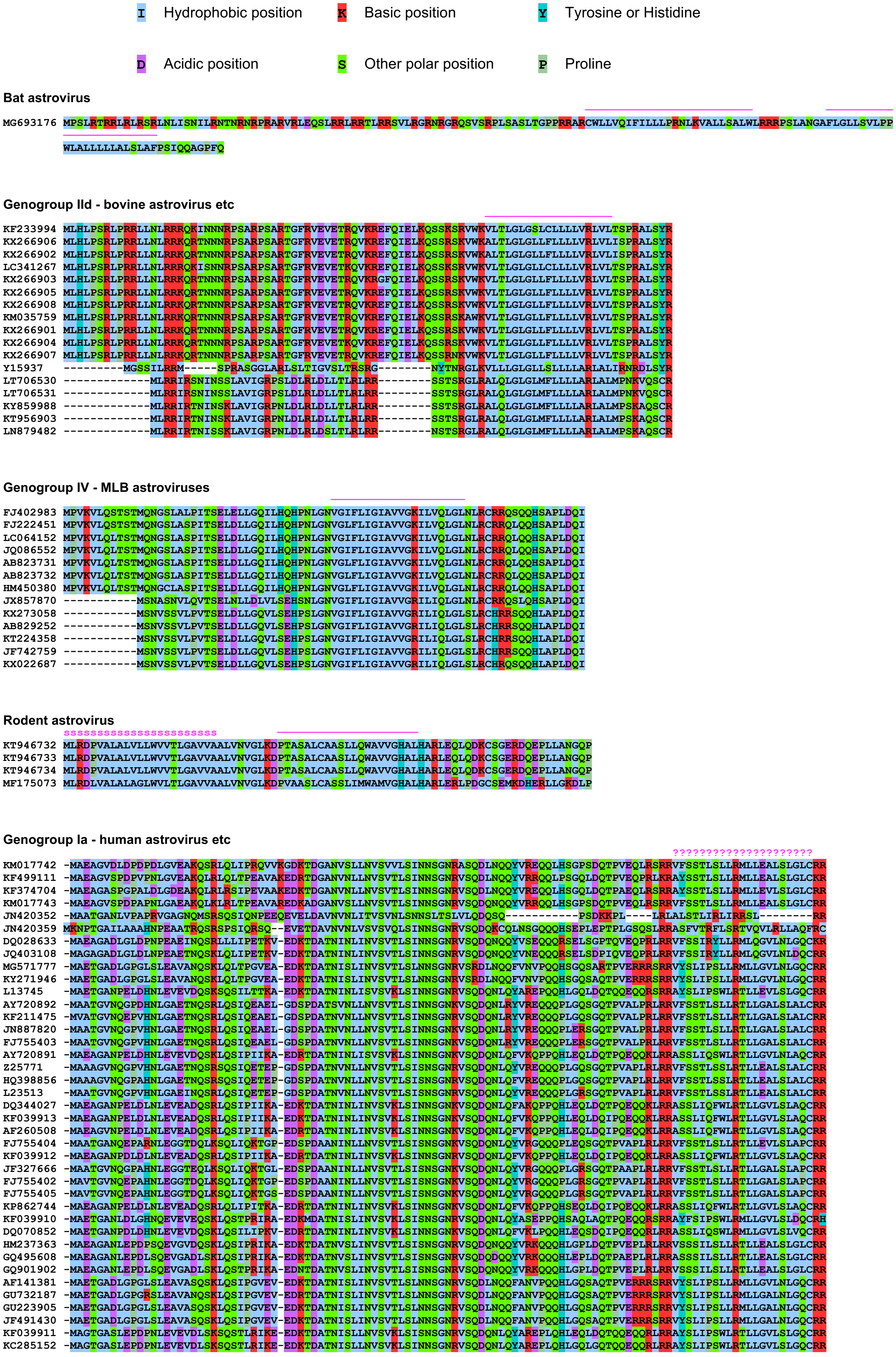
I Hydrophobic position
K Basic position
Y Tyrosine or Histidine
D Acidic position
S Other polar position
P Proline
Bat astrovirus
MG693176
Genogroup IId - bovine astrovirus etc
KF233994
KX266906
KX266902
LC341267
KX266903
KX266905
KX266908
KM035759
KX266901
KX266904
KX266907
Y15937
LT706530
LT706531
KY859988
KT956903
LN879482
Genogroup IV - MLB astroviruses
FJ402983
FJ222451
LC064152
JQ086552
AB823731
AB823732
HM450380
JX857870
KX273058
AB829252
KT224358
JF742759
KX022687
Rodent astrovirus
KT946732
KT946733
KT946734
MF175073
Genogroup Ia - human astrovirus etc
KM017742
KF499111
KF374704
KM017743
JN420352
JN420359
DQ028633
JQ403108
MG571777
KY271946
L13745
AY720892
KF211475
JN887820
FJ755403
AY720891
Z25771
HQ398856
L23513
DQ344027
KF039913
AF260508
FJ755404
KF039912
JF327666
FJ755402
FJ755405
KP862744
KF039910
DQ070852
HM237363
GQ495608
GQ901902
AF141381
GU732187
GU223905
JF491430
KF039911
KC285152

Genogroup Ic - California sea lion astrovirus etc

```
                                                                                         ?????????????????
JN420351 MKSLLRSTRVGLAANPVDGRNLAAGLRTSRSQLIQSQRSRDELDHVVGLVNVSLNLSNVTLTNQVQQDQDLQFLSGPQQRLALWVRILLAILSLRCVC
JN420354 MKSLLRSTLVDLAANPVAGQSHAAGLRMSRSQLIQSQNRSDEPDHVVGLVNVSLRLSDVSLTVQESQDQGHLLVREQPPRLGLLARILLALLSLRCVC
JN420357 MKSLLRSTPVDLAVSPVDDHSHVVAPKMSRSQSIQNLKRSDEPDHVVGLVNVSLTLSNVSLTVQELQDQDLPLGREQRRPLALLARILLALLSLRCAC
JN420358 MKSLLRSTPVDLAVSPVDDHSHVVAPKMSRSQSIQNLKRSDEPDHVVGLVNVSLTLSNVSLTVQELQDQDLPLGREQRRPLALLARILLALLSLRCAC
JN420356 MKSLLRSTPVDLAANPVGGQSHVAALRMSRSQSILNQRSSDEVDHVVGLINVSLQLSNVNLTVQELQDPEPLLDRQLQPHWARLARILLAILSLKCVC
```

Genogroup Ib - canine astrovirus etc

```
                                                                                    ?????????????????
KX599351 MSPLRLKPPEQNQHPPGANPGGETGMSKSLSTHNQSQTRGDETDLTIVVARELSLSLNGNSIRQESQDQNQQSPKLLHLLWVLLARILRVRLS
KX599349 MLPLRLNPPGQSQHPPGVNPGDGTGASRSLSTHNQGQIEGDETDLTIVVARELRLSLNGSSIRLESQDQDQQSPRLLHLLWGLLARILRVR--
KX599353 MLPLRLNPPGQSQHPPGVNPGDGTGMSKSLSTHNQGQIEGDETDLTIVVARELRLSLNGSSIRLESQDQNQLSPRLLHLLWELLARILRVQ--
KX756441 MLPLRLNPPVQNQHPPGANPGAGTGMSKSLSIHNQEQTEGDETDLTIVVARELRLSLNGSSIKLELQDQGQQSPRLLHLLWELLARILRVR--
MF973501 MSPSRLNPPVQTITPPGANPGDVTGMLRSQSIPNHKK-DADETDLTIVVAREMSLSLNGNLIRQELQDQNQQSPKLLHLLWGLLARILRVRLI
KP404150 MLPLRLNPPGQNQHPPGANPGAETGMSKSLSTHNQGQTEGDETDLTIVVARELRLSLNDSSIKLESQDQGQRLPKLLHLLWGLLARILRAQ--
KP404149 MSPLRLKPPEQNQHPLGANPGGGTGMSRSQSIHNPKQIGGDETDLTIVVARELRLSLNDSSIKLESQDQGQRLPKLLHLLWELLARILRVQ--
MF973500 MLPSRLNPPVQIITPPGASPGDVTGMLRSQSIPNHKK-GADETDLTIVVARELSLSLNGNSIRQELQDQNQQSPRLLHLLWGLLARILQVRLN
KX599350 MLPLRLKPPGQSQHPPGVNPGDGTGASRSLSTHNQGQIEGDETDLTIVVARELRLSLNGSSIRLESQDQDQQSPRLLHLLWGLLARILRVR--
KX599352 MLPSRLNPPVQTTTPPGASPGGVTGMLKLQSIPNHKK-DADETDLTIVVAKELSLSLNGNSIRQELQDQNQQSPKLLHLLWGLLARILRVRLN
```

Genogroup III - marmot astrovirus

```
         sssssssssssssssssssssssss
KY855439 MRRLPSPLRLLVVVLGGFLAGCAVGELDRRNNNQTYNQPLLQPLLVELLQYPMLLALVVGTVLGVVATLLVAQLTRQSRPPLERLVRTRETTLRQNL
KY855441 MRRSPSPLRLLAVVLGGFLAGCAVGELERRGNNQTHSQPLLQPLLVELLQYPMLLALVVGTVLGVVATLLVVQLTRQSRLPLERLVRTRETTLRQSL
KY855442 MRRSPSPLRLLAVVLGGFLAGCAVGELERRGNNQTHSQPLLQPLLVELLQYPMLLALVVGTVLGVVATLLVVQLTRQSRLPLERLVRTRETTLRQSL
KY855440 MRRSPSPLRLLAVVLGGFLAGCAVGELERRGNNQTHSQPLLQPLLVELLQYPMLLALVVGTVLGVVATLLVVQLTRQSRLPLERLVRTRETTLRQSL
```

Genogroup IIId - porcine astrovirus 4 etc

```
KY940075 ----MSNLKWSRLLRRCLTVVVGVAAGALIAYLQLLQPLLGELKLLNHLSGLVVGAIIGLAILISRLAPSSDRK----
LC201606 ----MSNPRWLRLRRRLLVVVVGVAAGAILAHLHLVRPLLGRWQFLDNLVGLLAGALIGLAILLNPAAPSSDKG----
LC201613 ----MSNPRWSRLLRRLLVVVVGVAAGGLLAHLRLVRPLLGGSPLLDNLVGLAVGAVIALALLVNRLLAASGKESQQH
KU764484 ----MSNPRWLRPLRRLLVVVVGAAAGAFLAHLHLIRPLLGRLPLLDNLVELLVGVAIALVNLAVRRLGALGKK----
KU764485 ----MSNPRWLRPLRRLLVVVVGAAAGAFLAHLHLIRPLLGRLPLLDNLVELLVGVAIALVNLAVRRLGALGKK----
KU764486 ----MSNLRWLRPLRRLLVVVVGVAAGALLAHLHLIRPLLGRLPLLDNLVEILVGVAIALVNLAVRHLGASDKK----
JF713713 ----MSSLRWSRLLRRCLTVVVGVAVGALIAYLQLLQPLLGELKLLSHLLGLAVGAIIGLAILINRVVPSSNKK----
LC201605 ----MSNPRWSRLLRRLLVVVVGAAAGVLLAHLRIIQPLLGKLPLLDNLLGLTVGAIIALAMLVNRLLPASDRE----
KX060809 ----MSNPRWSRLPRRLLVVVVGVAAGALLAHLQLTRPLLGRLPLLDNLVGLAVGAVIALALLVNRLLAASGNGSQQH
JQ340310 ----MSNPRWSRLRRRLLVVVVGVAAGALLAQLHLVRPLLGRWQFLDNLVGLLVAALIGLAILINPAAQPSNKE----
LC201609 ----MSNPRWLRLLRRLLVVVVGVAAGALLAHLRLARPLLGRLPLIDNLAGLVVGAVIALALVVNRLLEESDKGSQQH
KY933398 ----MSNPMWLRLLRRLLVVVVGVAAGGLLAHLRLVRPLLGRLPLIDNLVGLLVGAVLSLAILLSRVTRCTHKE----
LC201604 ----MSNLRWSRLLRRLLVVVVGVAAGALIAHLKLLQPLLGKLPLLDNLIGLVVGAVIALALLANRVLPSSNKG----
JX556692 ----MSNLRWSRLLRRCLTVVVGVAVGALIAYLQLLQPLLGELKLLNHLLGLAVGAIIGLAILINRVAPSSNKK----
LC201608 MTKTMSNPRWLRPLRRLLVVVVGVAAGALLAHLHLTKLLLGGSPLLDKLVGLIVGAVIALAILVGRHLGASGKE----
LC201603 ----MSNPLWLRLLRRFLTVVVGAAAGVLIAHLNLVQPLLGRSPLLDNLLGFAVGVVIGLAILTNKVLTSLSKK----
LC201600 ----MSNPRWSRLLRRLLVVVVGVAVGGILAHLRLVRPLLGGLPLLDNLVGLAVGAVIAMALLVNRLLVASDKGSQQH
LC201601 ----MSNPRWSRLLRRLLVVVVGVAVGGILAHLRLVRPLLGGLPLLDNLVGLAVGAVIAMALLVNRLLVASDKGSQQH
LC201602 ----MSNPRWSRLLRRLLVVVVGVAVGGILAHLRLVRPLLGGLPLLDNLVGLAVGAVIAMALLVNRVLPSSNKG----
KX033447 ----MSNPRWSRLRRRLLVVVVGVAAGALLAQLHLVRPLLGRWQFLDNLVGLLVVALIGLAILINPAAQHSGKQ----
KY214437 ----MSNPRWLRLLRRLLVVVVGVAVGGLLAHLRLVRPLLGRLPLIDNLAGLVVGAVIALALVVNRLLGVSGKGSQQH
KX060808 ----MSNLRWSRLLRRCLTVVVGVAAGVLIAHLNLVQPLLGKLPLLDNLVGLAVGAVIALVFLINRVLPSSSKE----
LC201610 ----MSNLKWSRLLRRCLTVVVGVAAGVLIAHLNLVQPLLGKLPLLDNLVGLAVGAVIALVLLINRVLPSSNKG----
LC201611 ----MSNLKWSRLLRRCLTVVVGVAAGVLIAHLNLVQPLLGKLPLLDNLVGLAVGAVIALVLLINRVLPSSNKG----
LC201612 ----MSNLKWSRLLRRCLTVVVGVAAGVLIAHLNLVQPLLGKLPLLDNLVGLAVGAVIALVLLINRVLPSSNKG----
```

Genogroup IIIa - murine astrovirus etc

```
         sssssssssssssssssssssssss
MF175075 MPRPSLLQLSLVAVVGGLAGALYGAALQALLTPQQRQQLFGLFFAAIPGLAVIAAGVGLLSGRLLARLSRRLRRPSERLARTRVIRSSLRWQRSSTRR
JX544743 MPRPSLLQLSLVAVVGGLAGALYGAALQALLTPQQRQQLFGLFFAALPGLAVIAAGVGMLSGRLLARLFRRLRRPSERLARTRAIRSSLRWQRSSTQR
JX544744 MPRPSLLQLSLVAVVGGLAGALYGAALQALLTPQQRQQLFGLFFAAIPGLAVIAAGVGMLSGRLLARLSRRLRRPSERLARTRAIRSSLRWQRSSAQR
KT946731 PRLPSPLSLAIVAV-GGVVGGAVLAQLDRLLQQQGQLPH--LVLAAVPGLAIGLAGATLVA-KLLPPSRKQSQQHWEPWDPIRAIP-----------
KT946730 ----------MVAV-SGLTGGAALAALEKLLQPQGLPPF--LLLLAVPGLVVVGLSAGLVAER-LTRSTKPSRRHSVP-------------------
KT946728 ----------MVAV-SGLTGGAALAALEKLLQPQGLPPF--LLLLAVPGLVVVGLSAGLVAER-LTRSTKPSRRHSVP-------------------
KT946729 ----------MVAV-SGLTGGAALAALEKLLQPQGLPPF--LLLLAVPGLVVVGLSAGLVAER-LTRSTKPSRRHSVP-------------------
KT946726 ----------MVAV-SGLTGGAALAALEKLLQPQGLPPF--LLLLAVPGLVVVGLSAGLVAER-LTRSTKPSRRHSVP-------------------
KT946727 ----------MVAV-SGLTGGAALAALEKLLQPQGLPPF--LLLLAVPGLVVVGLSAGLVAER-LTRSTKPSRRHSVP-------------------
```

* KT946731 lacks the ORFX AUG codon - possibly a defective sequence

Genogroup III - Rodent astrovirus

```
         ssssssssssssssssssssssssssssssss
KT946735 MPRLRSPLPRLVALVSGLAVGLWCAGIVKATGLPQQQQLFLHALATPGLVGFALALSVLVGMLQTPSPPRSPLHLVP
KT946736 MPRLRSPLPRLVALLSGLAVGLWCAGTVKATGLPQQQQLFLHALATPGLVGFALALSVLVGMLQTPSPPRSLLHSVP
```

Genogroup III - Bovine astrovirus

```
                                                             ????????????????????
LC047800 MAGRIGTLREWWSNLLQATNRHRPPPVGDVPDAELSMSTLIQ-DPHNSRRDSVPVQACACALTGVAAGVNKIRASFRYVRRSPQP---
KP264970 MVGRVGTPREWWSNLLQAINRHKRRRVRDANDGKLSMSMSTH--GLSHRTDSEQMLGCACALTGVLEGVKLPRVSLLSIRR-------
LC047798 MVGRTGTPHEWWSSLLQAINRQRRPRALDVRDGQQSMSMSTL--DLSQRRNSAPALACVCGLTAVVAGVNKVRASIKYIKR-------
LC047790 MAGRTATLRKWWCSLLQAINRL-PRCDLDAPDGPLSMSMSTQTEEILTETPMAPSLTCGCGLTAVFAGVNQIRASMKFTKPLQPPSAP
```

Genogroup IIIb - bovine astrovirus etc

```
LC047799 MDQLLHKVEQIPSSSSRG-RDEPDGGDSKIK---LGCLPFRLRLRVPGV-VSVR--VRALSTRRLSQHLAQWARIIRRQ-------
LC047789 MDQLLHKVEQIPNSSSRG-RDEPDGGDSKIK---LGCLPFRLRLRVPGV-VSVR--VRALSTRRLSQHLAQWARIIRRQ-------
LC047794 MDQLLHKVEQIPNSSSRG-RDEPDGGDSKIK---LGCLPFRLRLRVPGV-VSVR--VRALSTRRLSQHLAQWARIIRRQ-------
LC047795 MDQLLHKVEQAPNSSSRG-KDEPDGGDSKIR---LGCLPFKLRLRVPGV-VSVR--VRALSTRRLSQHLAQWARIIRRQ-------
LC047801 MDQLPHKVEQAPNSSSRG-KDEPDGGDSKIR---LGCLPFKLRLRVPGV-VSVR--VRALYTRRLSQHLAQWARTIRRQ-------
LC047788 MDQLPQQVGRPNSSSNRE-GDEPDGGDSRIR---LGCLPFRMQVRVPGV-VSVR--VRALCTSRLSRRLERWARIIQIK-------
LC047791 MDQPPHQVGRPNNSSSREEGDELDGGEGKLQ---LGCHPFRLRLRIPGV-ASVR--IRELSISGSSRRLGRLAQIILNKLKLN---
LC047792 MDQLPHQAGQPNSSSSREEGDEIDGGEGRFQ---LGCLPFRLRLRIPGV-ASVR--IRELSTSGLSQRSERLAQIILNRLKLN---
HQ916314 MDQLPPKVEAASQDSNN--SSVVDGAEQDHRPRSCGCFPTKLRVA-GGG-LGFHGSITVWWSRRLSQLSVRSAPMAQAQ-------
HQ916316 MDQLPPKVGAANQDNNS--SSAVDGAGQGRQPRWCGCYPSKIRVA-GGG-FGLRGSVTVWFTRRLSQLSAR---------------
MG660832 MEQLPTRQELSEDG-----VDGGTGETAKRRRSTCGCYPTKLRVS-AGI-LAARASVTVWFSRRSTQPSVQLDPTEVNRLNASLRA
KM822593 MDQLPHQVGQPNNNSSRGERDEIDGGEGKLQ---LGCLPFRLRLRVPGV-ASVR--IRELSTSELSQRLGRLARIIQTK-------
LC047797 MDQLPPKAEGSKLVCH---ADAVDGCKCKRCKH-RGCLPIG-RIRLSGAFLAARMSITVSFIRRSLRRSGP---------------
HQ916313 MEQLPTRQELPEDS-----VDGGAGGTTRRRRSTCGCYPTKLRFS-ARI-LAARASVTVWFSRRSTQPSVQLDPTEVSRLNASSRA
KJ620979 MEQLPTRQELSEDS-----VDGGTRGTTGRRRSTCGCYPTELRVS-AGV-LAARASVTVWFSRRSTQPSVQLDQTEVNRLNANSRA
KJ620980 MEQLPTRQELSEDS-----VDGGTRGTTGRRRSTCGCYPTKLRVS-AGV-LAARASVTVWFSRRSTQPSVQLDQTEVNRLNANSRA
HQ916317 MEQLPTRQELSEDS-----VDGGTGGTTKRRRSTCGCYPTKLRVS-AGI-LAARASVTVWFSRRSTQPSVQLDPTEVNRLNASSRA
LC047787 MEQLQPKVGSGKDSSKLH-ADDVDGCKCRRCRH-RGCLPVR-RIRLAGVFLAVRMSITASFTRRSRRRSGP---------------
LC047796 MEQLPTRQELLEDS-----ADGGAGGITRPRRSTCGCYPTKLRFS-AGF-LAARASVTVWFSRRSIQPSVQLDQTEVNRLNASLRA
```

Genogroup IIIc - porcine astrovirus 2 etc

```
                                                 ???????????????????
LC201590 --MAPARIRRAHPGPSRQEGEET--GRNH---KSISGCWPTKLRVSAGFLAARVSITAWFSRRSTQHSAPSDQMEASRLSVS---------
KP747573 --MAPPRIRRSLPEQIRQEGEGT--GEDHS--RPWCGCYPTKVRVAGAILGLRASVTVWLSRRLSQLSGQSDPMGVDQSRQNSPSSSTPAQ
KY940077 --MAPARIRRALPELNRQGEEGT--GENR---RLTSGCLPIKIRLSAGVLAARVSVVVWFSRRSRRPLAQSDQMVVSKSSVK---------
KP982872 --MAPARIRRALPEPNRQEEEGT--GESHS--RPWCGCYPTKIRVAGAFLGLRASVTVWLSRRLSRLSEQLDPTARDRSRQSSLSCSTPAQ
LT898434 --MAPARIRRALPEHNRQGGEET--GGNR---RSTSGCWPVKIRLSAGILAARVSVVVWFSRRSRQPSAQSDQMVVTRSSVR---------
LC201588 --MAPARIRRAHPGLNRQDEGET--EENHS--KPSCGCYPTRIRVAGAILGLRASVTVWLSRRLSQPSGQSGPTELDRSRQSLQSYSIPAQ
KP759770 --MAPPRIRRALPEQIRQEGEGT--GENHN--RPWCGCYPTRFRVAGAILGFRASVTVWLSRRLSQLSGQSDPMGVGQSRQNSLSSSTPAQ
KY214438 --MAPPRIRRALPEQIGQEGGET--EGNHN--RPLCGCYPTRVRVAGAFLGLRASVTVWLSRGLSRLSARSGRMGQAK-------------
KR868724 --MAPPQIRRALPRQSRQEGGEV--EGNH---RLTSGCCPIKVRLSASFLAARASVIVWFSRKSIRPLEQLDQMAVNK-------------
KR868723 --MAPPRIRRALPRQDKQGEGET--GENR---RSISGCCPVKIRLSAGFLAAKVSVVVWFSRRSIRPLGQLDPMAVNKSSVR---------
KR868722 --MAPPRIRRALPRQDKQGEGET--GGNH---RLISGCCPIKIRLSARFLAARVSVVVWFSRRSTRPLGQLGPMAVSKSSVR---------
KR868721 --MAPPRIRRALPRQDKQGEGGT--EGNH---RLISGCCPIKIRLSARFLAARVSVVVWFSRRSTRPLGQLGPMAVSKSSVR---------
LC201593 --MAPPRIRRALPEPNRQGDEGT--REDHS--QSTCGCLPTKVRVAGAVLGFRASVTVWWHRGLSRLSGRSVPTDPGT-------------
KY940076 --MAPARIRRALPEPNKQGEEGTEQGDRP---GSTCGCFPIKVRVAAGFLGARVSVIAWFSRRSTPRLAQLGRMRAERLSVN---------
LC201592 --MAQPRIRRALPALGRDAGGGT--GENNNNQRSTCGCYPTKIRVAGAFLGLRASVTVWWSRGLSRLSGRLGPTGLEK-------------
LC201587 --MAPARIRRARPGPSRQEGEEV--GENHS--RPWCGCYPTRIRVAGAILGLRASVTVWLSRRLSRPSEQSGPMEPDRSRQNSLSCSIPVQ
LC201589 MEQLPPR-RGRLVRRQAIEEGGT--GENP---KSTSRCWPTKVRLSAACLAAKGSITVWFSRRSTQHLERSAQTEVERSSVS---------
LC201594 MDRLPPR-RGRLARQQATEEGETE-GDRS---RPLCGCYPTKIRVAGAVLGARASVTVWLSRRLSQLSAQLGPMVADQSRRSSPSS-----
LC201585 MEQLPPR-RGRLARQQA-EGGETEQGDSP---RSMCGCYPLKLRVAAGFLGARVSVVAWFSRRSTRRSAQSARMGVNK-------------
LC201586 MEQLPTR-RGRLARQQATEGEGIEQGDRP---RSTCGCFPVKIRVAAGFLGARVSVVALFSRRSTRLLAQSDPMGVNK-------------
KJ495986 MEQLPPR-RGRLARQQ-VGGEETEQGDNP---KSTCGCYPLKLRVAAGFLGARVSVVAWFSRRSTQRSAQSAQMVVTK-------------
JF713710 MEQLPPR-RGRLARQQAVGGEEIEQGDNP---RSTCGCYPLKLRVAAGFLGARVSVVAWFSRRSIQHSAQLVQMEVSK-------------
JF713712 MEQLPPR-RGRLERQQATEGGGIEQGDNH---NSTCGCFPVKIRVAAGFLGARVSVVALFSRRLTQPSARLDPTGVSK-------------
JX556690 MEQLPPR-RGRLARQQAIGEGETEQGDNH---RSMCGCYPLKLRVAAGFLGARVSVVAWFSRRSIQHSAQSVQTEVSK-------------
```

Supplementary Figure 9 | Sequences of putative XP proteins encoded in diverse astroviruses. Amino acid alignments of putative XP proteins in different astrovirus groups. Sequences were aligned with MUSCLE[1]. Sequences are listed in the same order as in the ORF1b phylogenetic tree (Supplementary Fig. 1). Amino acids are colour-coded according to their physicochemical properties. Transmembrane regions predicted by Phobius[5] are indicated with pink bars above the alignment. Potential transmembrane regions (hydrophobic regions scored below threshold by Phobius) are indicated with pink question marks. Signal peptides predicted by Phobius are indicated with pink "s"s. In all cases, predictions are based on the first sequence in each alignment.

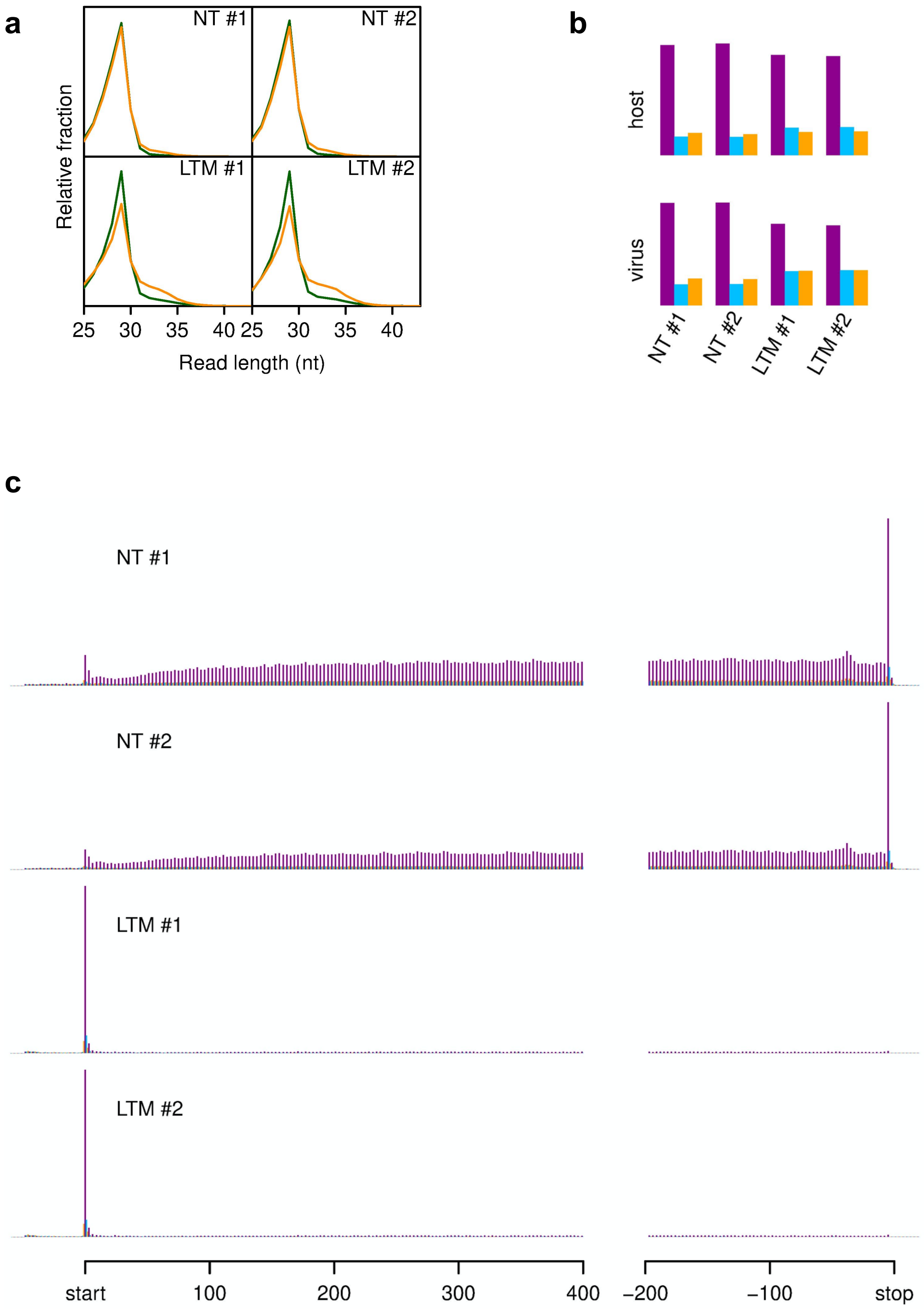

Supplementary Figure 10 │ Assessment of ribosome profiling quality. Cells were harvested at 12 hpi and either flash frozen with no pre-treatment (NT), or pre-treated with lactimidomycin for 30 min followed by flash freezing (LTM). **(a)** Relative length distributions for Ribo-Seq reads mapping to virus (orange) and host (green) mRNA coding regions. **(b)** Phasing of 5′ ends of RPFs (≥25 nt) that map to the viral ORFs (excluding dual coding regions) or host mRNA coding regions. **(c)** Histograms of approximate P-site positions of RPFs (≥25 nt) relative to annotated initiation and termination sites summed over all host mRNAs. See Supplementary Table 4 for read counts. Source data are provided as a Source Data file.

```
                 4330      4340      4350      4360      4370      4380      4390      4400      4410      4420
              ..|....|....|....|....|....|....|....|....|....|....|....|....|....|....|....|....|....|....|....|..
pAVIC1        ATGGCTAGCAAGTCCAATAAGCAGGTAACTGTTGAGGTCAGTAATAATGGCCGCAACAGGAGTAAATCAAGGGCCCGTTCACAATCTAGGGGCCGAGATA
CP             M  A  S  K  S  N  K  Q  V  T  V  E  V  S  N  N  G  R  N  R  S  K  S  R  A  R  S  Q  S  R  G  R  D
XP                                                           M  A  A  T  G  V  N  Q  G  P  V  H  N  L  G  A  E  I
pAVIC1-AUGm   ...............................................C....................................................
CP             M  A  S  K  S  N  K  Q  V  T  V  E  V  S  N  N  G  R  N  R  S  K  S  R  A  R  S  Q  S  R  G  R  D
XP                                                          (M)
pAVIC1-PTC1   ....................................................................................................
CP             M  A  S  K  S  N  K  Q  V  T  V  E  V  S  N  N  G  R  N  R  S  K  S  R  A  R  S  Q  S  R  G  R  D
XP                                                           M  A  A  T  G  V  N  Q  G  P  V  H  N  L  G  A  E  I
pAVIC1-2xPTC  ....................................................................................................
CP             M  A  S  K  S  N  K  Q  V  T  V  E  V  S  N  N  G  R  N  R  S  K  S  R  A  R  S  Q  S  R  G  R  D
XP                                                           M  A  A  T  G  V  N  Q  G  P  V  H  N  L  G  A  E  I
pAVIC1-PTC2   ....................................................................................................
CP             M  A  S  K  S  N  K  Q  V  T  V  E  V  S  N  N  G  R  N  R  S  K  S  R  A  R  S  Q  S  R  G  R  D
XP                                                           M  A  A  T  G  V  N  Q  G  P  V  H  N  L  G  A  E  I

                 4430      4440      4450      4460      4470      4480      4490      4500      4510      4520
              ..|....|....|....|....|....|....|....|....|....|....|....|....|....|....|....|....|....|....|..
pAVIC1        AATCAGTCAAGATTACAGTCAATTCAAGAAACAGAGCCAGGAGACAGCCCGGACGCGACAAACGTCAATCTTCTCAACGTGTCCGTAACATTGTCAATAA
CP            K  S  V  K  I  T  V  N  S  R  N  R  A  R  R  Q  P  G  R  D  K  R  Q  S  S  Q  R  V  R  N  I  V  N  K
XP             N  Q  S  R  L  Q  S  I  Q  E  T  E  P  G  D  S  P  D  A  T  N  V  N  L  L  N  V  S  V  T  L  S  I
pAVIC1-AUGm   ....................................................................................................
CP            K  S  V  K  I  T  V  N  S  R  N  R  A  R  R  Q  P  G  R  D  K  R  Q  S  S  Q  R  V  R  N  I  V  N  K
XP
pAVIC1-PTC1   .......G............................................................................................
CP            K  S  V  K  I  T  V  N  S  R  N  R  A  R  R  Q  P  G  R  D  K  R  Q  S  S  Q  R  V  R  N  I  V  N  K
XP             N  Q  *
pAVIC1-2xPTC  .......G.....A......................................................................................
CP            K  S  V  K  I  T  V  N  S  R  N  R  A  R  R  Q  P  G  R  D  K  R  Q  S  S  Q  R  V  R  N  I  V  N  K
XP             N  Q  *  R  *
pAVIC1-PTC2   ....................................................................................................
CP            K  S  V  K  I  T  V  N  S  R  N  R  A  R  R  Q  P  G  R  D  K  R  Q  S  S  Q  R  V  R  N  I  V  N  K
XP             N  Q  S  R  L  Q  S  I  Q  E  T  E  P  G  D  S  P  D  A  T  N  V  N  L  L  N  V  S  V  T  L  S  I

                 4530      4540      4550      4560      4570      4580      4590      4600      4610      4620
              ..|....|....|....|....|....|....|....|....|....|....|....|....|....|....|....|....|....|....|..
pAVIC1        GCAACTCAGGAAACAGGGTGTCACAGGACCAAAACCTGCAATATGTCAGAGAGCAACAGCAACCCTTGGGACGGTCGGGTCAAACACCAGTGGCACCACT
CP              Q  L  R  K  Q  G  V  T  G  P  K  P  A  I  C  Q  R  A  T  A  T  L  G  T  V  G  S  N  T  S  G  T  T
XP            S  N  S  G  N  R  V  S  Q  D  Q  N  L  Q  Y  V  R  E  Q  Q  Q  P  L  G  R  S  G  Q  T  P  V  A  P  L
pAVIC1-AUGm   ....................................................................................................
CP              Q  L  R  K  Q  G  V  T  G  P  K  P  A  I  C  Q  R  A  T  A  T  L  G  T  V  G  S  N  T  S  G  T  T
XP
pAVIC1-PTC1   ....................................................................................................
CP              Q  L  R  K  Q  G  V  T  G  P  K  P  A  I  C  Q  R  A  T  A  T  L  G  T  V  G  S  N  T  S  G  T  T
XP
pAVIC1-2xPTC  ....................................................................................................
CP              Q  L  R  K  Q  G  V  T  G  P  K  P  A  I  C  Q  R  A  T  A  T  L  G  T  V  G  S  N  T  S  G  T  T
XP
pAVIC1-PTC2   ..................................................................A.................................
CP              Q  L  R  K  Q  G  V  T  G  P  K  P  A  I  C  Q  R  A  T  A  T  L  G  T  V  G  S  N  T  S  G  T  T
XP            S  N  S  G  N  R  V  S  Q  D  Q  N  L  Q  Y  V  R  E  Q  Q  Q  P  *

                 4630      4640      4650      4660      4670      4680      4690      4700      4710      4720
              ..|....|....|....|....|....|....|....|....|....|....|....|....|....|....|....|....|....|....|..
pAVIC1        GAGATTGAGGCGTGTATTCTCCTCAACCCTGTCCTCGTTAAGGACGCTACTGGAAGCACTCAGTTTGGCCCTGTGCAGGCGCTAGGTGCACAGTATTCCA
CP             E  I  E  A  C  I  L  L  N  P  V  L  V  K  D  A  T  G  S  T  Q  F  G  P  V  Q  A  L  G  A  Q  Y  S
XP              R  L  R  R  V  F  S  S  T  L  S  S  L  R  T  L  L  E  A  L  S  L  A  L  C  R  R  *
pAVIC1-AUGm   ....................................................................................................
CP             E  I  E  A  C  I  L  L  N  P  V  L  V  K  D  A  T  G  S  T  Q  F  G  P  V  Q  A  L  G  A  Q  Y  S
XP
pAVIC1-PTC1   ....................................................................................................
CP             E  I  E  A  C  I  L  L  N  P  V  L  V  K  D  A  T  G  S  T  Q  F  G  P  V  Q  A  L  G  A  Q  Y  S
XP
pAVIC1-2xPTC  ....................................................................................................
CP             E  I  E  A  C  I  L  L  N  P  V  L  V  K  D  A  T  G  S  T  Q  F  G  P  V  Q  A  L  G  A  Q  Y  S
XP
pAVIC1-PTC2   ....................................................................................................
CP             E  I  E  A  C  I  L  L  N  P  V  L  V  K  D  A  T  G  S  T  Q  F  G  P  V  Q  A  L  G  A  Q  Y  S
XP
```

Supplementary Figure 11 │ Mutations introduced into pAVIC1. Nucleotide and CP (red) and XP (blue) amino acid sequences for pAVIC1, pAVIC1-AUGm, pAVIC1-PTC1, pAVIC1-2×PTC and pAVIC1-PTC2 mutants. Only nucleotide differences from wt pAVIC1 are shown for the mutants.

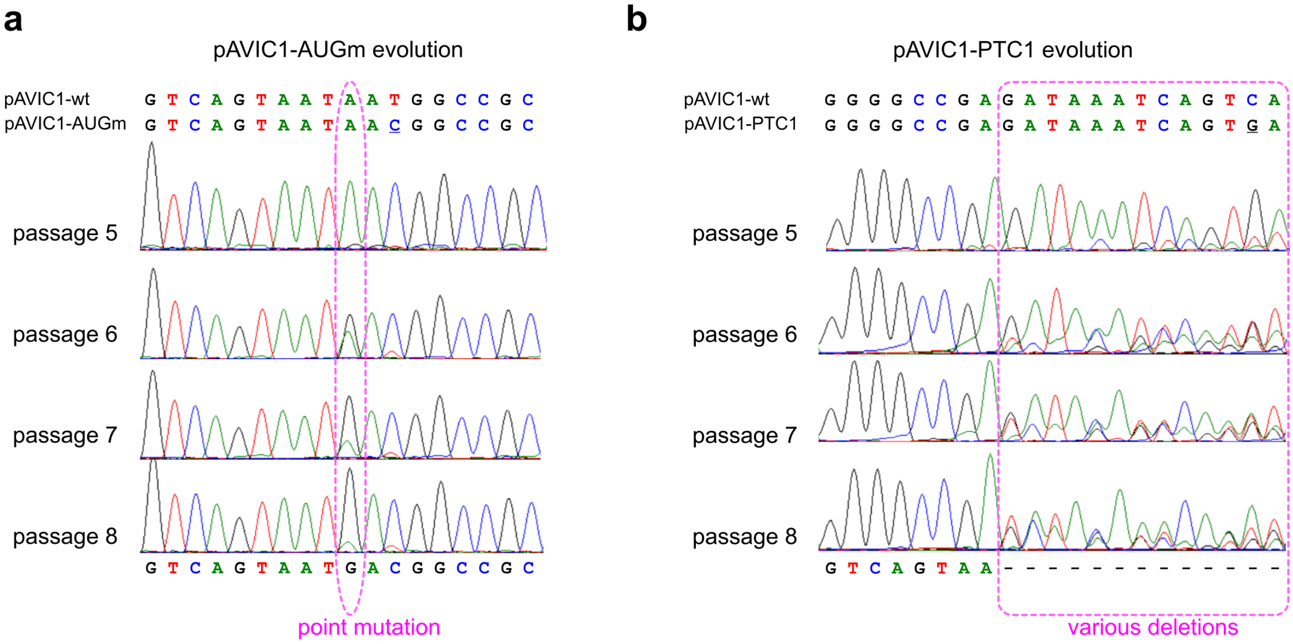

Supplementary Figure 12 | Evolution of XP knockout viruses. Sanger sequencing chromatograms of RT-PCR products obtained from passaged **(a)** pAVIC1-AUGm and **(b)** pAVIC1-PTC1. Evolution occurred via a pseudo-reversion (pAVIC1-AUGm) or 5- or 8-codon deletions (pAVIC1-PTC1). See Fig. 3c for individually sequenced cloned virus RT-PCR products.

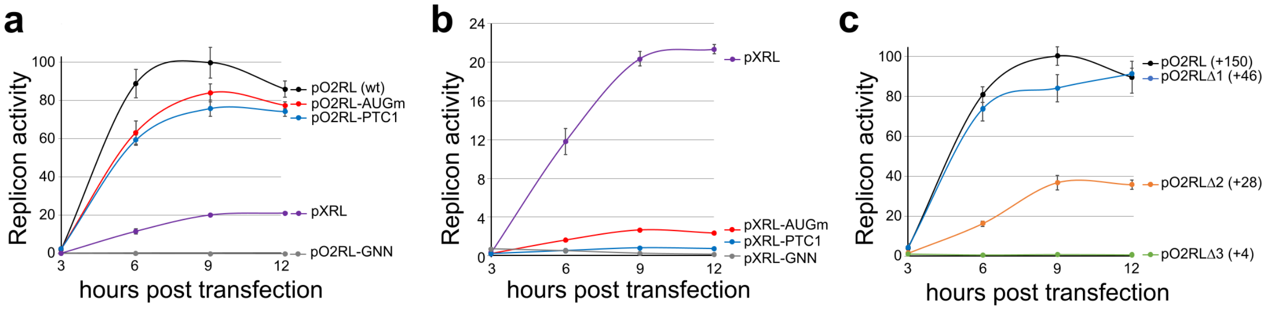

d Maximum replicon activity in BSR cells

replicon	wt	GNN	AUGm	PTC1	Δ1 (+46)	Δ2 (+28)	Δ3 (+4)
pO2RL	100 ± 3.0%	0.048 ± 0.002%	92.6 ± 3.6%	85.4 ± 2.6%	92.2 ± 1.9%	12.5 ± 0.1%	0.645 ± 0.027%
pXRL	13.7 ± 0.2%	0.111 ± 0.001%	2.45 ± 0.01%	0.747 ± 0.027%			

e Maximum replicon activity in Huh7.5.1 cells

replicon	wt	GNN	AUGm	PTC1	Δ1 (+46)	Δ2 (+28)	Δ3 (+4)
pO2RL	100 ± 8.1%	0.108 ± 0.004%	84.2 ± 4.6%	76.1 ± 4.3%	91.1 ± 2.9%	36.6 ± 3.6%	0.620 ± 0.104%
pXRL	21.4 ± 0.5%	0.274 ± 0.007%	2.68 ± 0.02%	0.828 ± 0.049%			

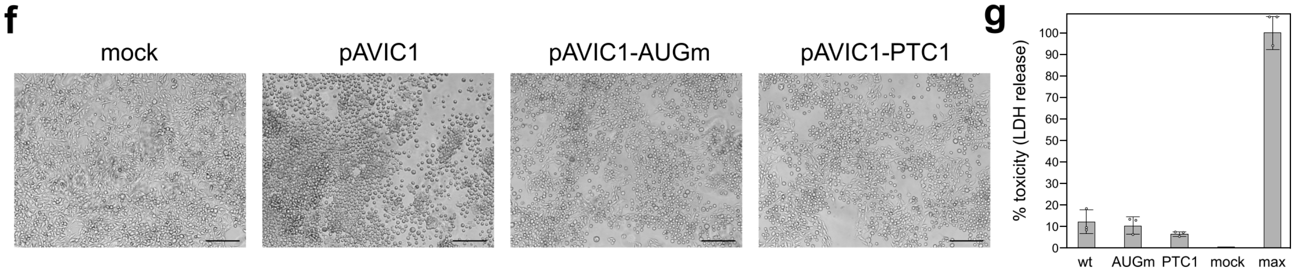

Supplementary Figure 13 | Supporting data for Figure 4. (a-c) Relative replicon luciferase activities representing translated product associated with activity of the subgenomic promoter, measured after RNA transfection of Huh7.5.1 cells (mean ± s.d.; *n* = 3 biologically independent experiments). Values are normalized so that the maximum wt value for ORF2-frame translation is 100%. **(d)** Maximum replicon activities obtained in the assays presented in Fig. 4c, d, f (BSR cells). **(e)** Maximum replicon activities obtained in the assays presented in Supplementary Fig. 13a, b, c (Huh7.5.1 cells). **(f)** Representative bright field images at 48 hpe of BSR cells electroporated with mock, pAVIC1, pAVIC1-AUGm or pAVIC1-PTC1 RNAs. Scale bars represent 100 µm. **(g)** Cell viability at 48 hpe for BSR cells electroporated with pAVIC1 or the indicated mutant RNAs, calculated as the ratio of released to total lactate dehydrogenase (LDH) activity (i.e. ($[LDH]_{test} - [LDH]_{negative}$) / ($[LDH]_{max} - [LDH]_{negative}$) (mean ± s.d.; *n* = 3 biologically independent experiments). Max = maximum LDH measured for fully lysed cells. Source data are provided as a Source Data file.

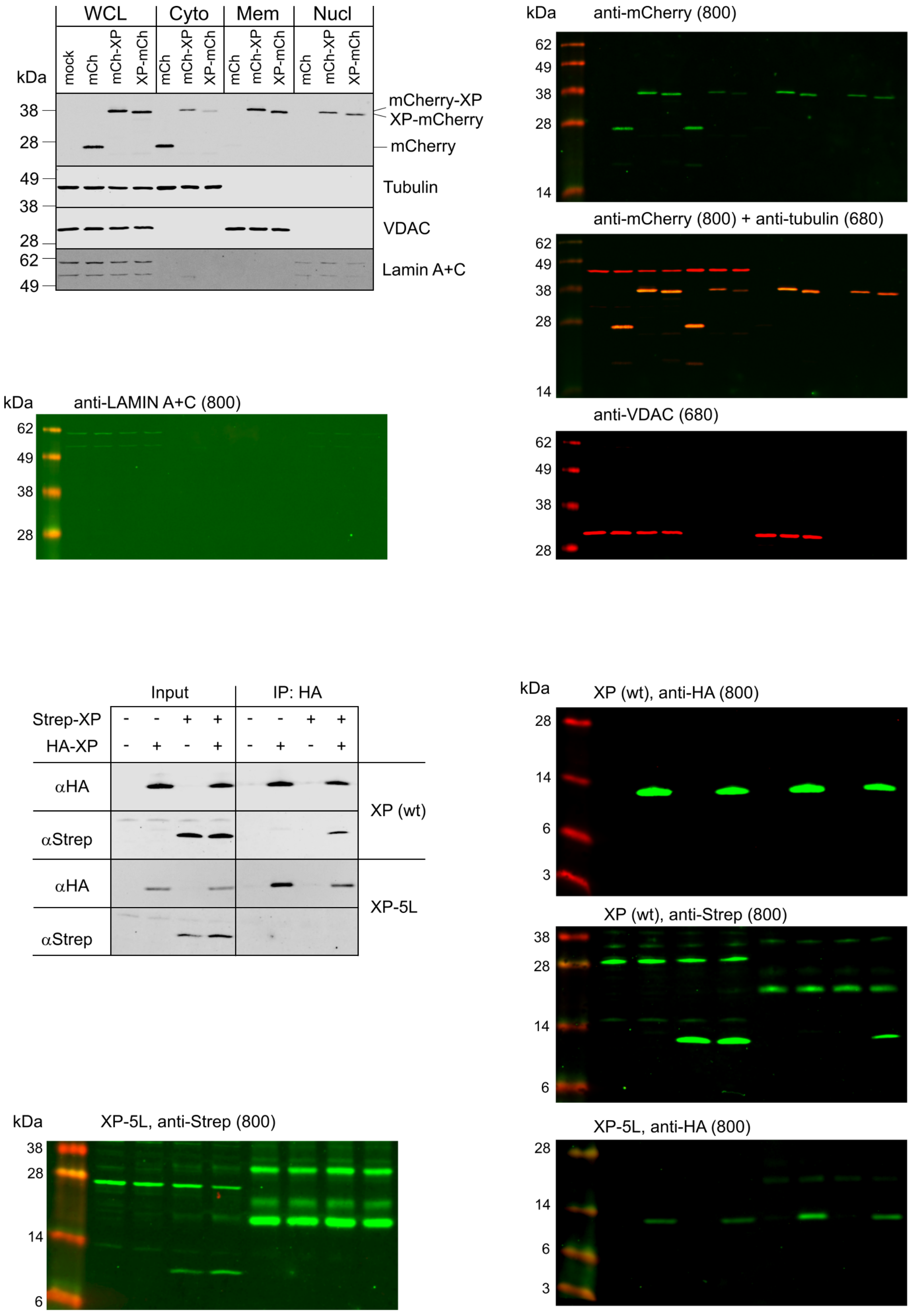

Supplementary Figure 14 | Original western blot scans for Fig. 5b,h.

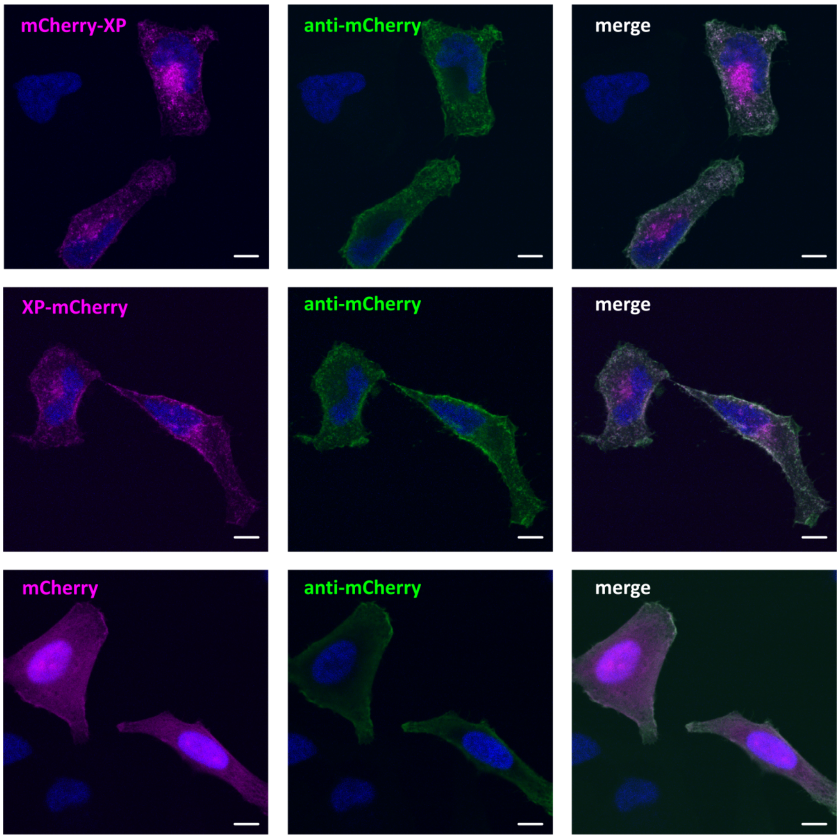

Supplementary Figure 15 │ Detection of XP-mCherry and mCherry-XP with anti-mCherry antibody. HeLa cells were electroporated with pCAG-mCherry, pCAG-XP-mCherry or pCAG-mCherry-XP. The plasma membrane (but not internal membranes) was selectively permeabilized with digitonin, cells were stained with anti-mCherry antibody followed by Alexa 488-labelled anti-rabbit IgG antibody, and visualized by confocal microscopy. The images are averaged single plane scans. Scale bars represent 10 µm.

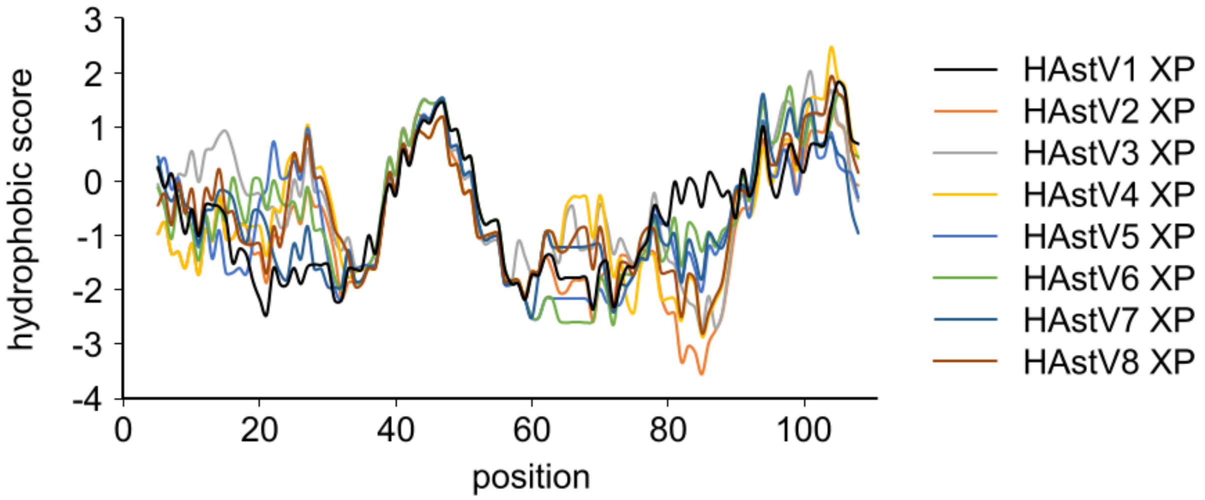

Supplementary Figure 16 │ Individual Kyte-Doolittle hydropathy plots for HAstV1-8 XPs. Hydropathy plots for HAstV1 pAVIC1 and representative HAstV2–8 sequences (see Fig. 1f) were predicted with protscale with a 9-aa window size (https://web.expasy.org/protscale; May 2019).

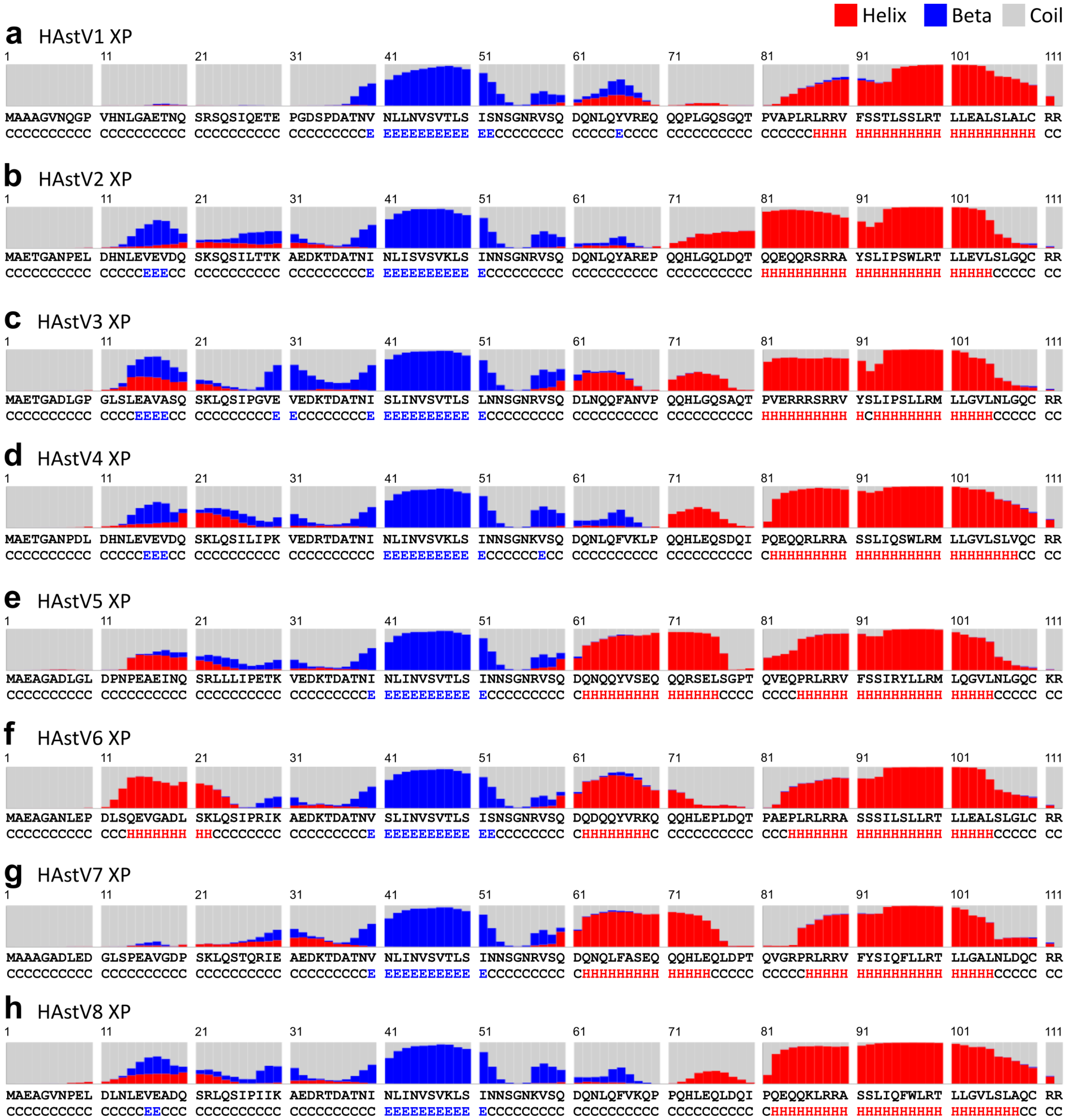

Supplementary Figure 17 | Predicted secondary structure of HAstV XPs. Protein secondary structures for representative sequences (see Fig. 1e) were predicted with RaptorX[6].

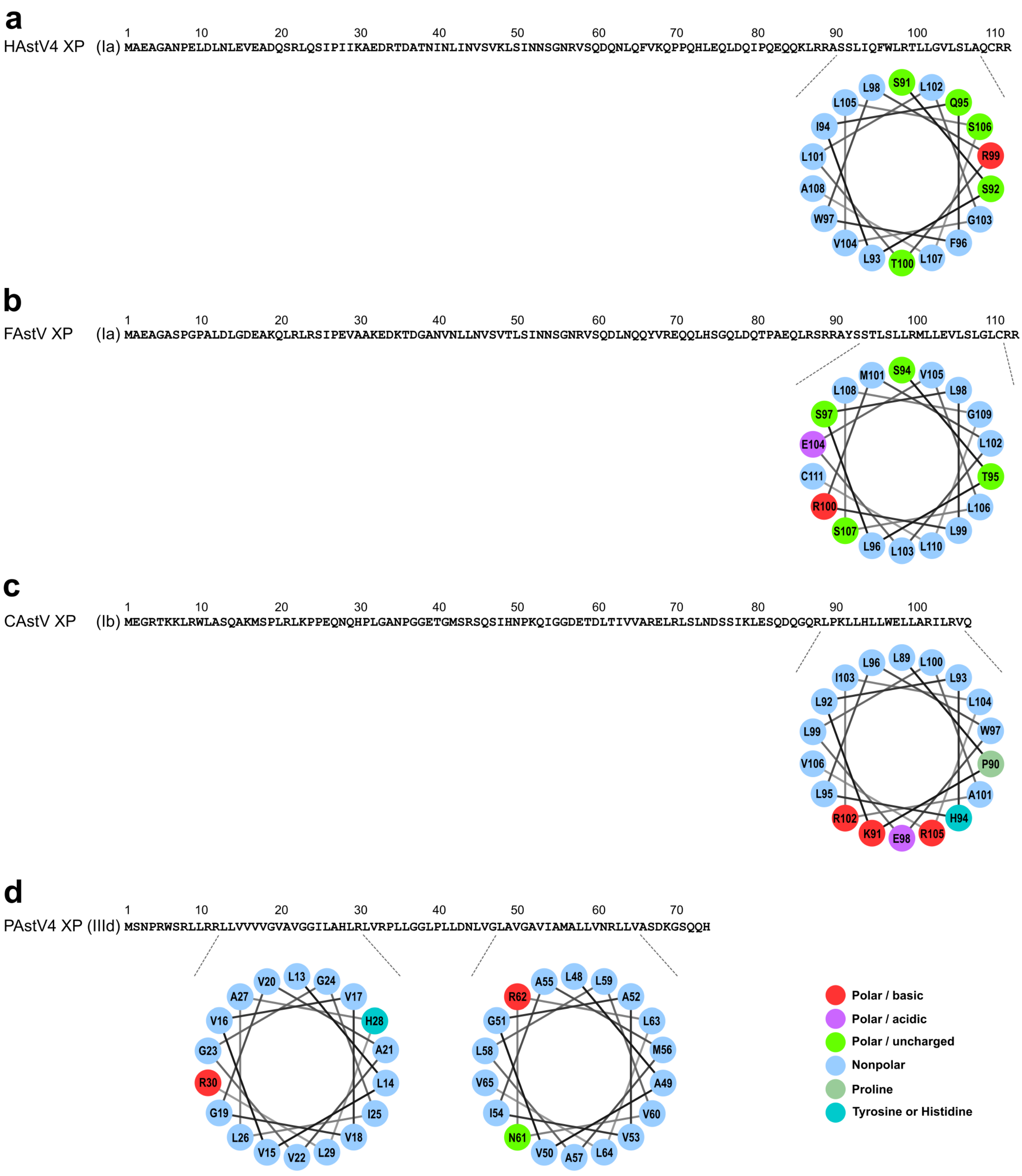

Supplementary Figure 18 │Helical wheel representations for TM regions of other astrovirus XPs used in Fig. 6e. TMs were predicted with Phobius[5] (http://phobius.sbc.su.se/) and helical wheels were created and analysed using Heliquest[7] (http://heliquest.ipmc.cnrs.fr; May 2019).

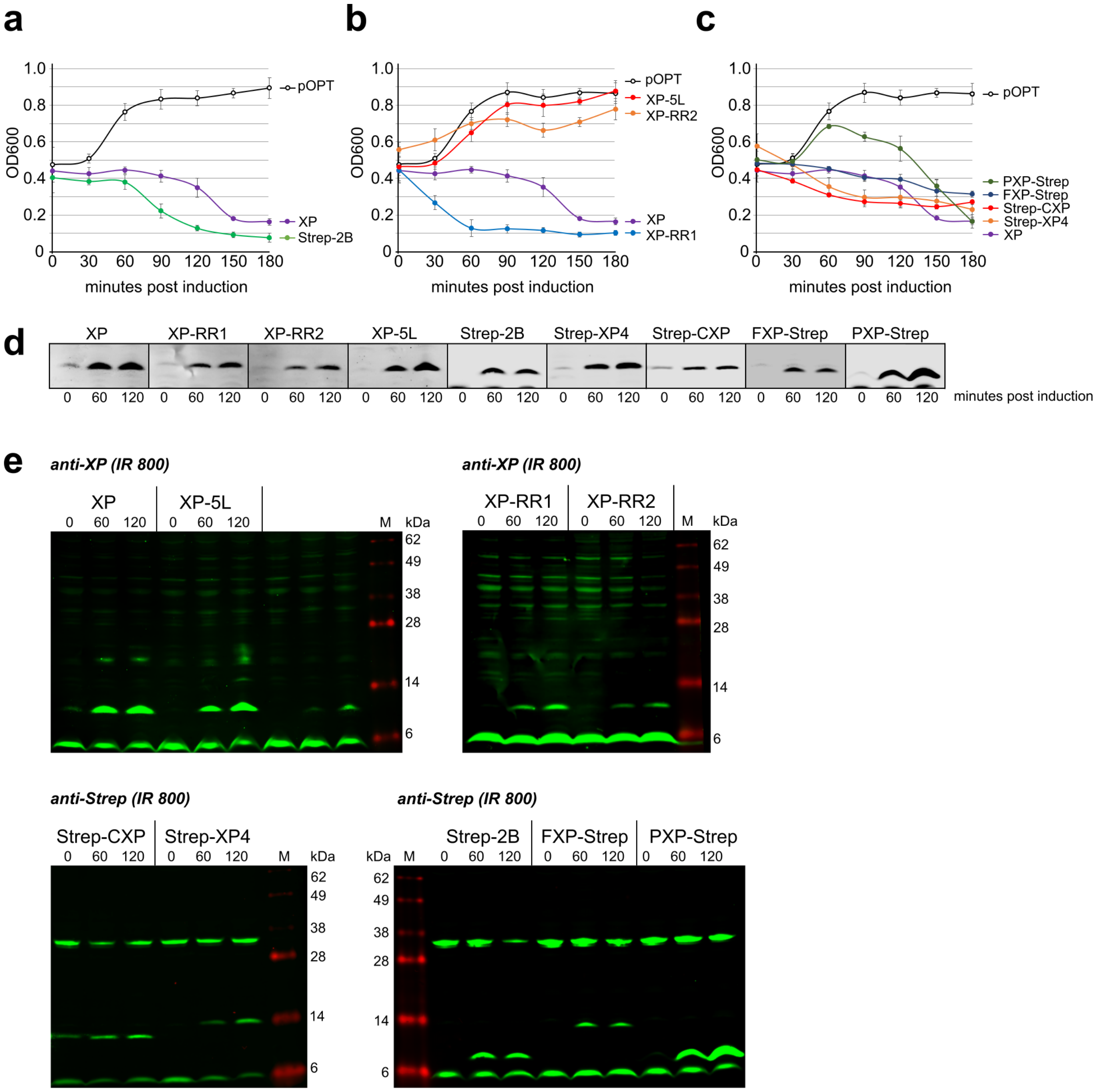

Supplementary Figure 19 │ Bacterial lysis assay for viroporin-like activity. (a-c) *E. coli* pLysS cells were transformed with pOPT constructs expressing the indicated proteins, grown to an optical density of 0.4 to 0.6, and then induced with IPTG. Optical densities were measured for induced and non-induced samples in triplicate over a time course of 180 min post induction. Graphs show means ± s.d. from *n* = 3 biologically independent experiments. **(d)** Non-induced, and 60 and 120 min post induction samples were also collected for protein detection by western blot. **(e)** Original western blot scans for (d). Source data are provided as a Source Data file.

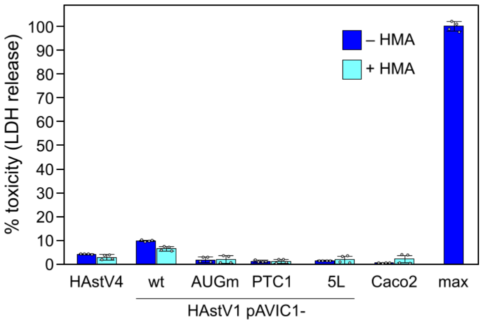

Supplementary Figure 20 │ Toxicity assay for HMA-treated astrovirus-infected cells. Caco2 cells were infected with the indicated viruses at MOI 0.2 in the presence (dark blue bars) or absence (light blue bars) of 5 μM hexamethylene amiloride (HMA) for 48 h. Supernatant was used to measure cell viability, calculated as the ratio of released to total lactate dehydrogenase (LDH) activity (mean ± s.d.; *n* = 4 biologically independent experiments). Max = maximum LDH measured for fully lysed cells. Source data are provided as a Source Data file.

Supplementary Table 1 │ XP and YP statistics in different astrovirus groups

Group	Protein	Number	Median mass (kDa)	Median pI	Median length (aa)	Predicted TM(s)
Bat astrovirus	XP	1	17.1	13.4	148	yes
Genogroup IId - bovine astrovirus etc	XP	17	10.7	12.7	91	yes
Genogroup IV - MLB astroviruses	XP	13	8.4	8.6	78	yes
Rodent astrovirus	XP	4	8.3	5.5	79	yes
Genogroup Ia - human astrovirus etc	XP	39	12.3	8.0	112	no
Genogroup Ic - California sea lion astrovirus etc	XP	5	10.8	7.9	98	no
Genogroup Ib - canine astrovirus etc	XP	10	10.1	9.7	91	no
Marmot astrovirus	XP	4	10.7	12.1	97	yes
Genogroup IIId - porcine astrovirus 4 etc	XP	25	7.6	12.5	70	yes
Genogroup IIIa - murine astrovirus etc	XP	9	6.5	12.1	64	yes
Rodent astrovirus	XP	2	8.0	12.5	77	yes
Bovine astrovirus	XP	4	9.1	11.5	82	no
Genogroup IIIb - bovine astrovirus etc	XP	19	8.3	11.3	73	no
Genogroup IIIc - porcine astrovirus 2 etc	XP	24	8.3	11.8	75	no
Genogroup IIc mamastrovirus 10 etc	YP	6	9.9	6.2	91	no

Supplementary Table 2 │ Accession numbers of the 415 astrovirus ORF2 sequences

AB000283 AB000284 AB000285 AB000286 AB000287 AB000288 AB000289 AB000290
AB000291 AB000292 AB000293 AB000294 AB000295 AB000296 AB000297 AB000298
AB000299 AB000300 AB000301 AB009984 AB009985 AB013618 AB025801 AB025802
AB025803 AB025804 AB025805 AB025806 AB025807 AB025808 AB025809 AB025810
AB025811 AB025812 AB031030 AB031031 AB037272 AB037273 AB037274 AB290149
AB308374 AB496913 AB823731 AB823732 AB829252 AB914705 AB914706 AF056197
AF117209 AF141381 AF248738 AF260508 AY179509 AY720891 AY720892 DQ028633
DQ070852 DQ344027 DQ630763 EF138823 EF138824 EF138825 EF138826 EF138827 EF138828
EF138829 EF138830 EF138831 EF583300 EU847144 EU847145 EU847155 FJ222451 FJ375759
FJ402983 FJ571065 FJ571066 FJ571067 FJ571068 FJ571070 FJ571071 FJ571072 FJ571073
FJ571074 FJ755402 FJ755403 FJ755404 FJ755405 FJ792842 FJ890351 FJ890352 FJ890355
FJ973620 FM213330 FM213331 FM213332 GQ267696 GQ405855 GQ405856 GQ405857
GQ415660 GQ415661 GQ415662 GQ495608 GQ502193 GQ891990 GQ901902 GQ914773
GU223905 GU376736 GU562296 GU732187 GU985458 HM045005 HM237363 HM447045
HM447046 HM450380 HM450381 HM450382 HM756258 HM756259 HM756260 HM756261
HQ398856 HQ623147 HQ623148 HQ647383 HQ668129 HQ668143 HQ916313 HQ916314
HQ916315 HQ916316 HQ916317 JF327666 JF491430 JF713710 JF713711 JF713712 JF713713
JF729316 JF742759 JF742760 JF755422 JN052023 JN088537 JN193534 JN420351 JN420352
JN420353 JN420354 JN420355 JN420356 JN420357 JN420358 JN420359 JN592482 JN887820
JQ081297 JQ086552 JQ340310 JQ403108 JQ408745 JX087963 JX087964 JX087965 JX544743
JX544744 JX544745 JX544746 JX556690 JX556691 JX556692 JX556693 JX684071 JX684072
JX857868 JX857869 JX857870 KC285152 KC342249 KC609001 KC692365 KC915034
KC915035 KF039910 KF039911 KF039912 KF039913 KF157967 KF211475 KF233994 KF374704
KF417713 KF499111 KF668570 KF787112 KF859964 KJ476832 KJ476833 KJ476834 KJ476835
KJ476836 KJ476837 KJ476838 KJ495986 KJ495987 KJ495991 KJ495992 KJ495993 KJ495994
KJ495996 KJ495999 KJ496001 KJ571486 KJ620979 KJ620980 KJ656124 KJ920196 KJ920197
KM017741 KM017742 KM017743 KM035759 KM358468 KM401565 KM822593 KP264970
KP404149 KP404150 KP404151 KP404152 KP663426 KP747573 KP747574 KP759770 KP862744
KP942582 KP942583 KP942584 KP942585 KP942586 KP942587 KP942588 KP942589 KP942590
KP942591 KP942592 KP942593 KP982872 KR349488 KR349489 KR349490 KR349491
KR868721 KR868722 KR868723 KR868724 KT224358 KT946726 KT946727 KT946728
KT946729 KT946730 KT946731 KT946732 KT946733 KT946734 KT946735 KT946736
KT956903 KT963069 KT963070 KT963071 KU764484 KU764485 KU764486 KX022687
KX033447 KX060808 KX060809 KX266901 KX266902 KX266903 KX266904 KX266905
KX266906 KX266907 KX266908 KX273058 KX599349 KX599350 KX599351 KX599352
KX599353 KX599354 KX645667 KX683863 KX756441 KY024237 KY024238 KY073229
KY073230 KY073231 KY073232 KY073233 KY214437 KY214438 KY271946 KY412124
KY412125 KY412126 KY412127 KY744137 KY744138 KY744139 KY744140 KY744141
KY855437 KY855438 KY855439 KY855440 KY855441 KY855442 KY859988 KY933398
KY933399 KY933670 KY940075 KY940076 KY940077 KY940545 L06802 L13745 L23513
LC047787 LC047788 LC047789 LC047790 LC047791 LC047792 LC047793 LC047794 LC047795
LC047796 LC047797 LC047798 LC047799 LC047800 LC047801 LC064152 LC201585 LC201586
LC201587 LC201588 LC201589 LC201590 LC201592 LC201593 LC201594 LC201595 LC201596
LC201597 LC201598 LC201599 LC201600 LC201601 LC201602 LC201603 LC201604 LC201605
LC201606 LC201608 LC201609 LC201610 LC201611 LC201612 LC201613 LC201615 LC201616
LC201617 LC201618 LC201619 LC201620 LC341267 LN879482 LT706530 LT706531 LT898424
LT898434 MF033385 MF033386 MF175073 MF175075 MF684776 MF973495 MF973496
MF973497 MF973498 MF973499 MF973500 MF973501 MG571777 MG660832 MG693176
S68561 U15136 Y08632 Y15937 Y15938 Z25771 Z33883 Z46658 Z66541

Supplementary Table 3 │ List of primers used

Primer	Purpose	Sequence
pAVIC-ATGminus_F	Site directed mutagenesis	CAGGTAACTGTTGAGGTCAGTAATAACGGCCGCAACAGGAGTAAATC
pAVIC-ATGminus_R		GATTTACTCCTGTTGCGGCCGTTATTACTGACCTCAACAGTTACCTG
pAVIC-PTC1_F		CTAGGGGCCGAGATAAATCAGTGAAGATTACAGTCAATTCAAGAAACAGAG
pAVIC-PTC1_R		CTCTGTTTCTTGAATTGACTGTAATCTTCACTGATTTATCTCGGCCCCTAG
pAVIC-PTC2_F		CTAGGGGCCGAGATAAATCAGTGAAGATAACAGTCAATTCAAGAAACAGAGCC
pAVIC-PTC2_R		GGCTCTGTTTCTTGAATTGACTGTTATCTTCACTGATTTATCTCGGCCCCTAG
pAVIC-PTC3_F		CAGAGAGCAACAGCAACCCTAGGGACGGTCGGGTCAAAC
pAVIC-PTC3_R		GTTTGACCCGACCGTCCCTAGGGTTGCTGTTGCTCTCTG
X-GS-mCh-F		GTTTGGCCCTGTGCAGGCGCGGATCCGTGAGCAAGGGCGAGGAGG
X-SG-mCh-R:		GAGGAGTGAATTCGAAGATCTTAAGTTACTTGTACAGCTCGTCCATGCC
mCh-GS-X-F		GGCAAAGAATTAATTAAGCCACCATGGTGAGCAAGGGCGAGGAG
mCh-GS-X-R		CTTGATTTACTCCTGTTGCGGCGGATCCCTTGTACAGCTCGTCCATGCC
pAVIC-5L_F		AGTCCTCGTCAAGGACGCTACAGGAAGCACACAGTTTGGCCCAGTGCAGGCGCTAGGTGCAC
pAVIC-5L_R		TGGGCCAAACTGTGTGCTTCCTGTAGCGTCCTTGACGAGGACTGGGTTGAGGAGAATACACGCC
XP-5L-1R		GCTTCGGATAGCGTCCTAGTCGAGGAAGTGGTTGAGGAGAATACACGCCTC
XP-RR-1R		CAGTAGCGTCCTTAACGAGGACAGGGTTGAGGAGAATACAGCAGCCAATCTCAGTGGTGCCACTGG
XP-2R-R-BamHI		CGCGGATCCTTATGCTGCGCACAGGGCCAAACTGAGTGCTTCCAGTAGCGTCCTTAACGAGGAC
pAVIC-GNN-F		GTTTATGGAAATAACAGGCTTTCTACAACACCTTCGG
pAVIC-GNN-R		GTAGAAAGCCTGTTATTTCCATAAACTACAGTGTCATAGGTC
4714-O2-2A-R	Cloning of replicon	GAGAAGGTCAAAATTCAAAGTCTGTTTCACCGGTGCCACAATTTTCTGTTTGTGTCTGGCTTCACCTAGCGCCTGCACAG
2A-RLuc-F		CAGACTTTGAATTTTGACCTTCTCAAGCTGGCGGGAGACGTCGAGTCCAACCCCGGGCCCGCTTCCAAGGTGTACGACCC
RLuc-TAG-6148-R		GCTGTGTTAGTTTTAAGTTCCACGTTACTACTGCTCGTTCTTCAGCACGC
4714-X-2A-R		GAGAAGGTCAAAATTCAAAGTCTGTTTCACCGGTGCCACAATTTTCTGTTTGTGTCTGGCTTCGCGCCTGCACAGGGCC
SINV_F_Aat2	SINV-repC cloning	CCGACAGATTGTTCGACGTCAAG
SINV_R_XhoI		TTCCCCTCGAGGAATTCCCTTTTTTTTTTTTTTTTTTTTTTTTTTTTTTTTTTGAAATG
CP_R_NdeBam		ATTAGGATCCATATATATACATATGTGCGGACCACTCTTCTGTCC
CP_F_NdeBam		tccgcaCATATGTATATATATGGATCCTAATCCGCTACGCCCCAATGATCCG
mCh-Nde-F		GGGAATTCCATATGGTGAGCAAGGGCGAGG
mCh-Bam-R		CGCGGATCCTTACTTGTACAGCTCGTCCATGCC
Strep-2B-NdeI-F	Cloning of 2B, XPs into pOPT, SINV-repC	GGGAATTCCATATGTGGTCACATCCTCAGTTCGAAAAGTCCGCCGGGATCAAGGACTATGTCGAAC
2B-BamHI-R		CGCGGATCCTTATTGGCGTTCAGCCATGGGTATTC
Strep-XP4-NdeI-F		GGGAATTCCATATGTGGTCACATCCTCAGTTCGAAAAGTCCGCCGCCGAAGCAGGAGCAAATCC
XP4-BamHI-R		CGCGGATCCTTAGCGCCTGCACTGGGCCAAAC
pCAG-StrXP-F-Pac	Cloning into pCAG vector	ATACCTTAATTAAGCCACCATGTCCGCCTGGTCACATCCTCAGTTCGAAAAGGCCGCAACAGGAGTAAATCAAG
pCAG-XP-R-Afl		ATATACTTAAGTTAGCGCCTGCACAGGGCC
pCAG-XP-f-Pac		ATACCTTAATTAAGCCACCATGGCCGCAACAGGAGTAAATCAAG
pCAG-XP-HA-R-Afl		ATATACTTAAGTTAAGCGTAATCTGGAACATCGTATGGGTAGGCGGAGCGCCTGCACAGGGCC

pCAG-XPStr-R-Afl		ATATACTTAAGTTACTTTTCGAACTGAGGATGTGACCAGGCGGAGCGCCTGCACAGGGCC
pCAG-HAXP-F-Pac		ATACCTTAATTAAGCCACCATGTCCGCCTACCCATACGATGTTCCAGATTACGCTGCCGCAACAGGAGTAAATCAAG
SINV-RT	Reverse transcription	GTTGAAGAATCCGCATTGCATGG
HAstV1-RT		TACTGCTGTAGCAATAAGGCCACG
SINV-qF	qPCR	GAAACAATAGGAGTGATAGGCA
SINV-qR		TGCATACCCCTCAGTCTTAGC
HAstV1-qF		TGCTATTGGTACTGTCATGGG
HAstV1-qR		GGTGTGAAATGGAATTGTGGG
hamGAPDH-qF		GGCAAGTTCAAAGGCACAGTC
hamGAPDH-qR		CACCAGCATCACCCCATTT

Supplementary Table 4 │ Host and virus read counts for different Ribo-Seq samples

drug	repeat	time point	total reads	host rRNA	host mRNA	vRNA
No treatment	NT #1	12 hpi	34,261,071	23,068,841	3,960,680	778,926
	NT #2	12 hpi	41,436,501	27,367,513	5,669,906	1,035,209
Lactimidomycin	LTM #1	12 hpi	11,621,251	6,024,566	625,793	177,015
	LTM #2	12 hpi	12,903,118	9,177,384	828,982	240,408

Supplementary References

1. Edgar, R. C. MUSCLE: a multiple sequence alignment method with reduced time and space complexity. *BMC Bioinformatics* **5**, 113 (2004).
2. Ronquist, F. *et al.* MrBayes 3.2: efficient Bayesian phylogenetic inference and model choice across a large model space. *Syst. Biol.* **61**, 539–42 (2012).
3. Yokoyama, C. C. *et al.* Adaptive Immunity Restricts Replication of Novel Murine Astroviruses. *J. Virol.* **86**, 12262–12270 (2012).
4. Firth, A. E. Mapping overlapping functional elements embedded within the protein-coding regions of RNA viruses. *Nucleic Acids Res.* **42**, 12425–39 (2014).
5. Käll, L., Krogh, A. & Sonnhammer, E. L. L. Advantages of combined transmembrane topology and signal peptide prediction-the Phobius web server. *Nucleic Acids Res.* **35**, (2007).
6. Wang, S., Li, W., Liu, S. & Xu, J. RaptorX-Property: a web server for protein structure property prediction. *Nucleic Acids Res.* **44**, W430–W435 (2016).
7. Gautier, R., Douguet, D., Antonny, B. & Drin, G. HELIQUEST: a web server to screen sequences with specific alpha-helical properties. *Bioinformatics* **24**, 2101–2 (2008).
