# Supplementary material for: A hidden gene in astroviruses encodes a viroporin
Source: Nat Commun. 2020 Aug 13;11:4070. doi: 10.1038/s41467-020-17906-x (PMC7426862; doi:10.1038/s41467-020-17906-x)
Supplement: Supplementary file 3 — Reporting Summary [file 41467_2020_17906_MOESM3_ESM.pdf]

nature research

Corresponding author(s): Andrew Firth + Valeria Lulla

Last updated by author(s): Jul 17, 2020

Reporting Summary

Nature Research wishes to improve the reproducibility of the work that we publish. This form provides structure for consistency and transparency in reporting. For further information on Nature Research policies, see Authors & Referees and the Editorial Policy Checklist .

Statistics

For all statistical analyses, confirm that the following items are present in the figure legend, table legend, main text, or Methods section.

n/a	Confirmed	
☐	☒	The exact sample size (*n*) for each experimental group/condition, given as a discrete number and unit of measurement
☐	☒	A statement on whether measurements were taken from distinct samples or whether the same sample was measured repeatedly
☐	☒	The statistical test(s) used AND whether they are one- or two-sided<br>*Only common tests should be described solely by name; describe more complex techniques in the Methods section.*
☒	☐	A description of all covariates tested
☒	☐	A description of any assumptions or corrections, such as tests of normality and adjustment for multiple comparisons
☐	☒	A full description of the statistical parameters including central tendency (e.g. means) or other basic estimates (e.g. regression coefficient) AND variation (e.g. standard deviation) or associated estimates of uncertainty (e.g. confidence intervals)
☐	☒	For null hypothesis testing, the test statistic (e.g. *F*, *t*, *r*) with confidence intervals, effect sizes, degrees of freedom and *P* value noted<br>*Give P values as exact values whenever suitable.*
☐	☒	For Bayesian analysis, information on the choice of priors and Markov chain Monte Carlo settings
☒	☐	For hierarchical and complex designs, identification of the appropriate level for tests and full reporting of outcomes
☒	☐	Estimates of effect sizes (e.g. Cohen's *d*, Pearson's *r*), indicating how they were calculated

Our web collection on statistics for biologists contains articles on many of the points above.

Software and code

Policy information about availability of computer code

Data collection	LI-COR ODYSSEY CLx, Leica LAS AF confocal microscop, Spectra Max i3x (Molecular Devices) microplate reader, ViiA 7 Real-time PCR system (Applied Biosystems). RNA-seq was performed on a NextSeq 500 instrument, samples were sequenced with single-read of 76 bases using the NextSeq 500 high Output Kit 75-cycles (Illumina).
Data analysis	MrBayes version 3.2.3; FigTree version 1.4.2 (http://tree.bio.ed.ac.uk/software/figtree/); MUSCLE version 3.8.31; Phobius (http://phobius.sbc.su.se/; Apr 2019); RaptorX (http://raptorx.uchicago.edu/; May 2019); SYNPLOT2 version 1; Heliquest (http://heliquest.ipmc.cnrs.fr; May 2019); protscale (https://web.expasy.org/protscale; May 2019); Image Studio version 5.2; code2aln version 1.2; EMBOSS version 6.6.0.0; TMHMM (http://www.cbs.dtu.dk/services/TMHMM/; Feb 2020); SOSUI (http://harrier.nagahama-i-bio.ac.jp/sosui/sosui_submit.html; Feb 2020); HHpred (https://toolkit.tuebingen.mpg.de/tools/hhpred; Jun 2019); ImageJ (https://imagej.nih.gov/ij/index.html); FASTX-Toolkit version 0.0.13 (http://hannonlab.cshl.edu/fastx_toolkit); bowtie1 version 0.12.9.<br>The SYNPLOT2, Ribo-Seq data analysis pipeline, and astrovirus sequence processing code are freely available in the GitHub repositories https://github.com/AndrewFirth12/synplot2, https://github.com/AndrewFirth12/RiboseqAnalysis, and https://github.com/AndrewFirth12/AstrovirusORFx, respectively.

For manuscripts utilizing custom algorithms or software that are central to the research but not yet described in published literature, software must be made available to editors/reviewers. We strongly encourage code deposition in a community repository (e.g. GitHub). See the Nature Research guidelines for submitting code & software for further information.

Data

Policy information about availability of data

All manuscripts must include a data availability statement. This statement should provide the following information, where applicable:

- Accession codes, unique identifiers, or web links for publicly available datasets
- A list of figures that have associated raw data
- A description of any restrictions on data availability

The source data underlying Figures 1,2,4,5,6 and Supplementary Figures 10, 13, 19, 20 are provided as a Source Data file.
The sequencing data reported in this paper (Fig. 2; Fig. S10) have been deposited in ArrayExpress (http://www.ebi.ac.uk/arrayexpress) under the accession number E-MTAB-8045.
Virus sequence data for comparative genomic analyses were obtained from the National Center for Biotechnology Information GenBank nucleotide database (https://www.ncbi.nlm.nih.gov/nucleotide/).

Field-specific reporting

Please select the one below that is the best fit for your research. If you are not sure, read the appropriate sections before making your selection.

[x] Life sciences [ ] Behavioural & social sciences [ ] Ecological, evolutionary & environmental sciences

For a reference copy of the document with all sections, see nature.com/documents/nr-reporting-summary-flat.pdf

Life sciences study design

All studies must disclose on these points even when the disclosure is negative.

Sample size	Most experiments were performed in triplicate to guard against spurious results. As biological significance is only ascribed when the observed effects are large, n = 3 is sufficient, except for confocal co-localization measurements (n = 12). No statistical methods were used to predetermine sample size. Sample sizes were chosen based on prior knowledge in the respective experiments and their intrinsic variability as performed in previous studies (Lulla et al., Nature Microbiology, 2019).
Data exclusions	After establishing protocols, no data were excluded from reported experiments.
Replication	Experiments were repeated at least to n = 3 to verify reproducibility. After establishing protocols, all attempts at replication were successful.
Randomization	No experimental groups were used in this study.
Blinding	The investigators were not blinded because collection and analysis of the presented data is not prone to bias. All experiments are precise (and generally quantitative) measurements of enzyme activity, protein labeling or protein expression levels and are not based on subjective assessments.

Reporting for specific materials, systems and methods

We require information from authors about some types of materials, experimental systems and methods used in many studies. Here, indicate whether each material, system or method listed is relevant to your study. If you are not sure if a list item applies to your research, read the appropriate section before selecting a response.

Materials & experimental systems

n/a	Involved in the study	
[ ]	[x]	Antibodies
[ ]	[x]	Eukaryotic cell lines
[x]	[ ]	Palaeontology
[x]	[ ]	Animals and other organisms
[x]	[ ]	Human research participants
[x]	[ ]	Clinical data

Methods

n/a	Involved in the study	
[x]	[ ]	ChIP-seq
[x]	[ ]	Flow cytometry
[x]	[ ]	MRI-based neuroimaging

Antibodies

Antibodies used	Alexa Fluor 488-conjugated secondary antibodies (Thermo Fisher; A21441 and A11015)<br>Alexa Fluor 568-conjugated donkey anti-rabbit antibody (Thermo Fisher, A10042)<br>Astrovirus 8E7 antibody, mouse monoclonal IgG (Santa Cruz Biotechnology, sc-53559)<br>Custom rabbit polyclonal antibody raised against XP peptide SNSGNRVSQDQNLQ (GenScript)<br>Anti-Strep mouse antibody (Abcam, ab184224)

	Anti-HA rabbit antibody (Abcam, ab20084)<br>Anti-tubulin (Abcam, ab15568)<br>Anti-VDAC1 (Abcam, ab14734)<br>Anti-LAMIN A+C (Abcam, ab133256)<br>Anti-TGN46 (BioRad, AHP500G)<br>Anti-GM130 (BD Biosciences, 610882)<br>Anti-mCherry (Abcam, ab167453)<br>Wheat Germ Agglutinin Alexa Fluor™ 488 Conjugate (WGA, Thermo Scientific, W11261)
Validation	The used antibodies were validated by commercial parties (GenScript) and the suppliers (Thermo Fisher, Santa Cruz Biotechnology, Abcam, BioRad, BD Biosciences) and/or previous literature reports for the used species and applications. Anti-HA, anti-Strep, anti-mCherry were also validated using overexpressing HeLa or Huh7.5.1 lysates.

Eukaryotic cell lines

Policy information about cell lines

Cell line source(s)	BSR cells (a single cell clone of BHK-21 cells, ATCC)<br>HeLa cells (ATCC, CCL-2)<br>Caco2 (ATCC, HTB-37)<br>Huh7.5.1 cells (Apath, Brooklyn, NY)
Authentication	For Caco2 cells, the RiboSeq data analysis confirmed the human origin of the cells line. HeLa (human), Huh7.5.1 (human) and BSR (hamster) cells have been authenticated to species level by the same method, but in other studies performed in our laboratory.
Mycoplasma contamination	All cells were mycoplasma tested (MycoAlertTM PLUS Assay, Lonza); BSR, Huh7.5.1 and Caco2 cells were also tested by deep sequencing.
Commonly misidentified lines<br>(See ICLAC register)	No commonly misidentified cell lines were used in this study except HeLa cells. HeLa cells were used only for localization and membrane topology studies of overexpressed XP fusion proteins; any possible misidentification would not affect the study.
